# HIRA and dPCIF1 coordinately establish totipotent chromatin and control orderly ZGA in *Drosophila* embryos

**DOI:** 10.1073/pnas.2410261121

**Published:** 2024-11-14

**Authors:** Guoqiang Zhang, Yaqi Miao, Yuan Song, Liangliang Wang, Yawei Li, Yuanxiang Zhu, Wenxin Zhang, Qinmiao Sun, Dahua Chen

**Affiliations:** ^a^Institute of Biomedical Research, Yunnan University, Kunming 650500, China; ^b^State Key Laboratory of Membrane Biology, Institute of Zoology, Chinese Academy of Sciences, Beijing 100101, China; ^c^School of Life Sciences, University of Chinese Academy of Sciences, Beijing 100049, China; ^d^Institute of Stem Cells and Regeneration, Chinese Academy of Sciences, Beijing 100101, China; ^e^Southwest United Graduate School, Kunming 650500, China

**Keywords:** early embryo, pioneer factor, zygotic genome activation, *Drosophila*

## Abstract

Early embryos undergo profound changes in their genomic architecture to establish the totipotent state, enabling pioneer factors to access chromatin and drive zygotic genome activation (ZGA). However, the mechanisms by which the totipotent state is established and properly interpreted by pioneer factors to allow orderly ZGA remain unknown. Here, we identify the H3.3-specific chaperone HIRA as a factor involving establishing totipotent-state chromatin in *Drosophila* early embryos. Through cophase separation with HIRA, the pioneer factor GAGA factor (GAF) efficiently binds to H3.3-marked nucleosomes to activate major-wave zygotic genes. Importantly, dPCIF1, a chromatin-associated protein, antagonized the GAF–HIRA interaction by competitively binding to HIRA, thereby restricting GAF on earlier chromatin and avoiding premature ZGA. Hence, the coordinated action of HIRA and dPCIF1 ensures sequential ZGA from the minor to major wave in early embryos. This study provides insights into understanding how a totipotent state is established and properly controlled during ZGA.

## Significance

In animals, establishing a totipotent chromatin state is essential for early embryogenesis. However, the mechanisms by which the totipotent chromatin is de novo established, enabling pioneer factors to precisely bind to target nucleosomal DNA, remain elusive. Here, we report that *Drosophila* HIRA plays a role in establishing totipotent chromatin in early embryos. H3.3-containing nucleosomes generated by HIRA act as markers allowing the pioneer factor GAGA factor (GAF) to access chromatin, thereby activating widespread zygotic transcription. Additionally, dPCIF1 functions as a surveillance factor to prevent premature zygotic genome activation (ZGA) by antagonizing GAF activity in the early embryo. Our study reveals a paradigm to understand how a totipotent state is properly established and regulated, ensuring orderly ZGA in early embryos.

In animals, early embryo development is initially supported by maternal factors (e.g., proteins and RNAs) until the zygotic genome is activated in a process called zygotic genome activation (ZGA) (1, 2). As maternal factors degrade, the zygotic genome begins controlling gene expression, a transition known as the maternal-to-zygotic transition (3). Although the zygotic genome is transcriptionally silent during the very early developmental period, the embryo undergoes profound changes in its genomic architecture, thereby reprogramming the chromatin from a germ cell to a totipotent state (4, 5). This enables pioneer factors, specialized maternal transcription factors, to bind to DNA within nucleosomes, promoting early zygotic gene expression and creating accessible chromatin by reorganizing and remodeling chromatin (3, 6–8). This enables the recruitment of downstream transcription factors to access the target DNA and activate the gene expression necessary for embryonic development. Despite this, questions remain about how the totipotent chromatin state is established and maintained for pioneer factors to function effectively.

In *Drosophila*, early embryogenesis is characterized by 13 rounds of rapid and synchronous nuclear divisions (NCs). Zygotic transcription is initiated at approximately NC8 and gradually activates until NC13, and the zygotic genome becomes widely activated at the final division, NC14 (3). While Zelda acts as a critical pioneer factor in accessing the early embryonic genome by binding to TAGteam sites for the earliest wave of ZGA (9–12), GAGA factor (GAF), encoded by the *Trithorax-like* (*Trl*) gene, as another pioneer factor via binding to GA dinucleotides activates widespread zygotic transcription and remodel the chromatin accessibility landscape (13, 14). The sequential and coordinated actions of Zelda and GAF are important to control orderly ZGA. However, little is known about how this orderly ZGA program is established and maintained. Notably, enrichment of GA dinucleotides in the embryonic genome remains accessible even in the absence of maternal Zelda (9, 15). This suggests that GAF accesses chromatin and activates major-wave ZGA, at least in part through a Zelda-independent mechanism (13). However, GAF is highly expressed in the early embryo, but it performs its major function at a later stage, suggesting an uncharacterized surveillance system restricting GAF to prematurely access early chromatin.

HIRA is an evolutionarily conserved histone chaperone that specifically deposits the histone H3.3 variant (H3.3) into nucleosomes in a replication-independent manner (16, 17). Genetic studies have revealed that depletion of H3.3 or HIRA causes developmental defects in many species (18–24). In *Drosophila*, HIRA plays a role in the formation of the diploid zygote (25); moreover, in early embryo, the incorporation of H3.3 on chromatin increases with each cycle of nuclear division, particularly doubling between NC10 and NC13, indicating a potential role of H3.3 in ZGA (26). However, whether and how this histone variant and its chaperone HIRA contribute to ZGA remains unclear. In this study, we focused on chromatin-associated protein dPCIF1 and unexpectedly found that dPCIF1 directly interacts with HIRA to antagonize premature activation of zygotic genes induced by the pioneer factor GAF. Mechanistically, HIRA assembles sequence-specific H3.3-containing nucleosomes and enables GAF to access chromatin by binding to HIRA, and subsequently activating major-wave ZGA at a later stage when dPCIF1 is expressed at a low level. At the earlier stage, when levels of dPCIF1 are high, dPCIF1 competitively binds to HIRA to antagonize the GAF–HIRA function, allowing orderly ZGA. Our data provide mechanistic insights into understanding ZGA regulation.

## Results

### dPCIF1 Acts as a Chromatin-Associated Factor to Regulate *Drosophila* Embryogenesis.

**Fig. 1.**
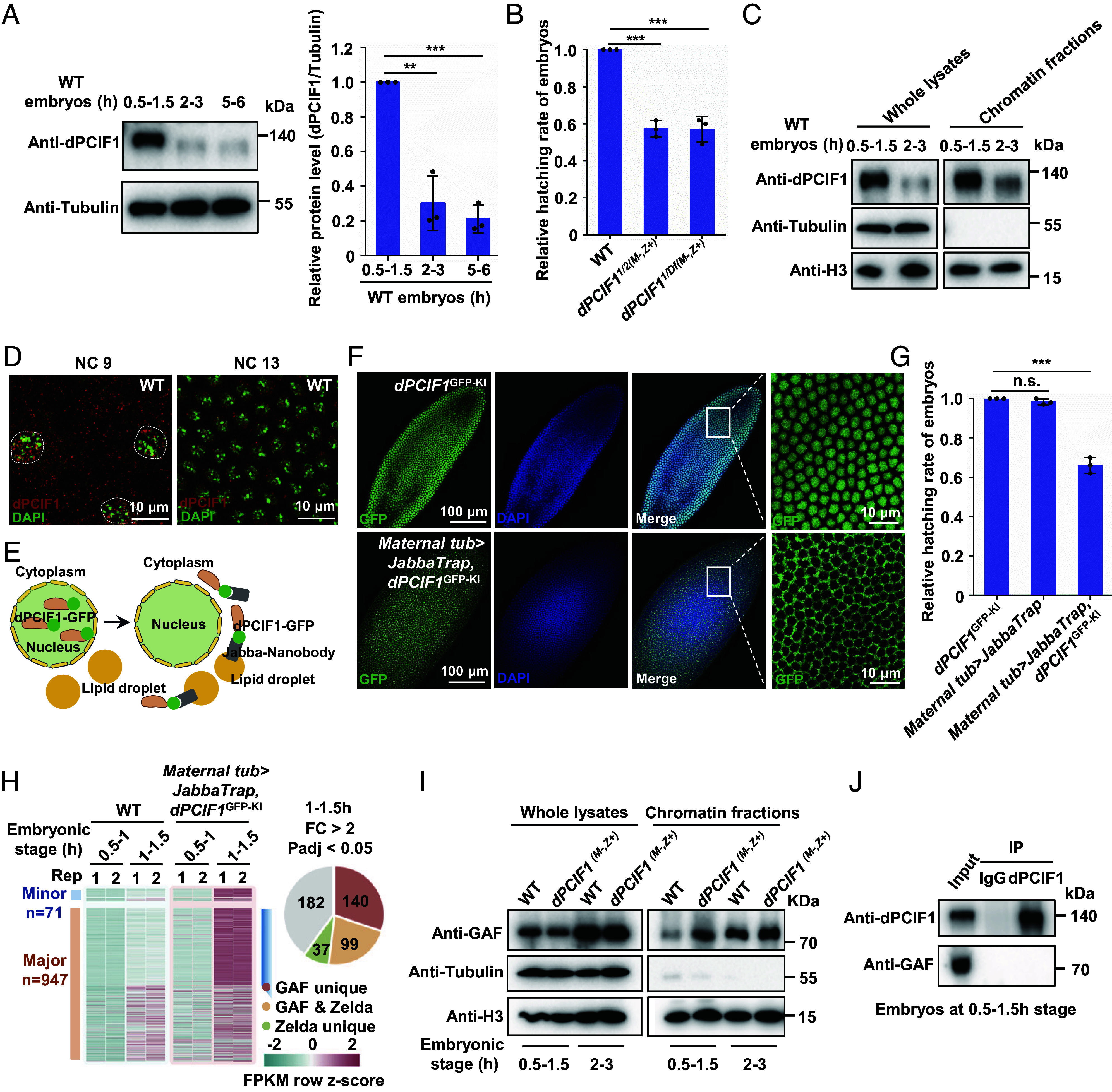
dPCIF1 acts as a chromatin-associated factor that regulates *Drosophila* embryogenesis. (*A*) Western blot showing the dynamic expression of dPCIF1 in wild-type embryos at three different stages. The *Right* panel shows the quantitative intensity of bands boxed from three biological replicates. (*B*) Bar plot showing that loss of maternal dPCIF1 protein causes a significant embryonic lethal phenotype. (*C*) Western blot showing the abundance of dPCIF1 in whole lysates and chromatin fractions extracted from wild-type embryos. (*D*) Immunostaining showing the localization of dPCIF1 in wild-type embryos at indicated stages. (Scale bars, 10 μm.) (*E*) Schematic representation of the JabbaTrap system. (*F*) Immunostaining showing a cytosol-localization of dPCIF1-GFP in mislocalized dPCIF1 mutant embryos. (Scale bars, as indicated.) (*G*) Bar plot showing the relative hatching rate of mislocalized dPCIF1 mutant embryos. (*H*) *Left* panel, heatmap showing expression of minor and major wave zygotic transcripts in wild-type and mislocalized dPCIF1 mutant embryos at two different stages before major-wave ZGA. *Right* panel, pie chart showing that ~52% of ectopically expressed major wave genes are GAF targets. (*I*) Western blot showing the abundance of GAF in whole lysates and chromatin fractions from wild-type and *dPCIF1* maternal mutant embryos. (*J*) Co-IP showing the interaction of endogenous dPCIF1 with GAF in wild-type embryos. Error bars indicate mean ± SD (n = 3). **P < 0.01, ***P < 0.001, n.s., not significant.

We characterized the function of *CG11399* (referred to here as *dPCIF1*) because its homologue PCIF1 catalyzes the formation of cap-specific m6Am in mammalian mRNA (27, 28). Consistent with previous findings (27, 29), mass spectroscopic analysis revealed no signal of m6Am modification in the whole body and multiple tissues of flies (*SI Appendix*, Fig. S1*A*). We examined the dPCIF1 pattern at various embryonic stages and found high expression of dPCIF1 at the early (0.5 to 1.5 h) embryonic stage, and then its expression level was dramatically reduced at late stages (2 to 3 h) (Fig. 1*A*), raising a possibility that dPCIF1 might be involved in regulating *Drosophila* embryogenesis. We employed the CRISPR/Cas9 method to generate two null mutant alleles for the dPCIF1 protein (*SI Appendix*, Fig. S1 *B* and *C*). Using these two mutant alleles and the dPCIF1 deficiency line, we generated *dPCIF1* maternal mutant embryos and performed hatching rate analysis. As shown in Fig. 1*B*, loss of maternal dPCIF1 protein consistently caused a significant embryonic lethal phenotype. Next, we sought to understand the molecular mechanism underlying the action of dPCIF1 in early embryos. To this end, we generated a GFP-tagged dPCIF1 knock-in allele, *dPCIF1*^GFP-KI^, using the CRISPR/Cas9 technique (*SI Appendix*, Fig. S1*D*). Genetic and western blot assays revealed that flies carrying homozygous *dPCIF1*^GFP-KI^ displayed normal germline and embryonic development, as indicated by immunostaining and embryo hatching rate analysis (*SI Appendix*, Fig. S1 *E* and *F*), and GFP knock-in did not change the level of endogenous dPCIF1 in knock-in flies (*SI Appendix*, Fig. S1*G*). Considering the high affinity of the anti-GFP antibody against GFP, we carried out coimmunoprecipitation (co-IP) assays followed by mass spectroscopic analysis using *dPCIF1*^GFP-KI^ early embryos (0.5 to 1.5 h) (*SI Appendix*, Fig. S1*H*). In this assay, we identified a large number of dPCIF1-interacting proteins. As shown in *SI Appendix*, Fig. S1*I*, consistent with previous findings (30), Pol II subunits were abundant in dPCIF1 immuno-precipitants. Moreover, we found that histone proteins, such as H2A/H2Av, H2B, H3/H3.3, and H4, were associated with dPCIF1. Chromatin fractionation assays (Fig. 1*C*) showed a significant proportion of dPCIF1 in the chromatin fraction, although it was also detected in the cytoplasm at the early (0.5 to 1.5 h) stage (Fig. 1*D*). These findings together suggest that dPCIF1 is a chromatin-associated protein. To ask whether the chromatin-associated dPCIF1 regulates early embryonic development, we sought to remove dPCIF1 from the chromatin by performing a JabbaTrap assay using the *dPCIF1*^GFP-KI^ strain. The JabbaTrap system traps GFP-tagged nuclear/chromatin-localized proteins in lipid droplets in the cytoplasm, thereby inactivating their nuclear/chromatin function in embryos (31) (Fig. 1*E*). As shown in Fig. 1*F*, nuclear/chromatin localization of GFP-tagged dPCIF1 was blocked when the JabbaTrap system was introduced into homozygous *dPCIF1*^GFP-KI^ embryos. Moreover, the hatching rate assays revealed that Jabba trapping of dPCIF1 led to a significant embryonic lethal phenotype (Fig. 1*G*). Collectively, our results support that dPCIF1 functions as a chromatin-associated factor to regulate embryonic development.

### dPCIF1 Maintains Normal ZGA by Antagonizing Function of Pioneer Factor GAF.

After identifying dPCIF1 as a chromatin-associated factor that regulates embryonic development, we examined whether maternal loss of chromatin-associated dPCIF1 affects the early chromatin state and zygotic gene expression. For this purpose, we collected wild-type and mislocalized dPCIF1 mutant embryos at early stages and then performed immunostaining assays using anti-RNA Pol II-Ser5p and anti-H3K27ac antibodies. RNA Pol II-Ser5p and H3K27ac are markers of active gene expression (32–34). In wild-type embryos, signals of RNA Pol II-Ser5p and H3K27ac were observed at NC6 and NC8, respectively (35) (*SI Appendix*, Fig. S2 *A* and *B*). However, in the dPCIF1-mislocalized mutant, RNA Pol II-Ser5p and H3K27ac signals were detected earlier at NC5 and NC7, respectively (*SI Appendix*, Fig. S2 *A* and *B*). These findings suggest that loss of dPCIF1 leads to premature activation of early chromatin.

**Fig. 2.**
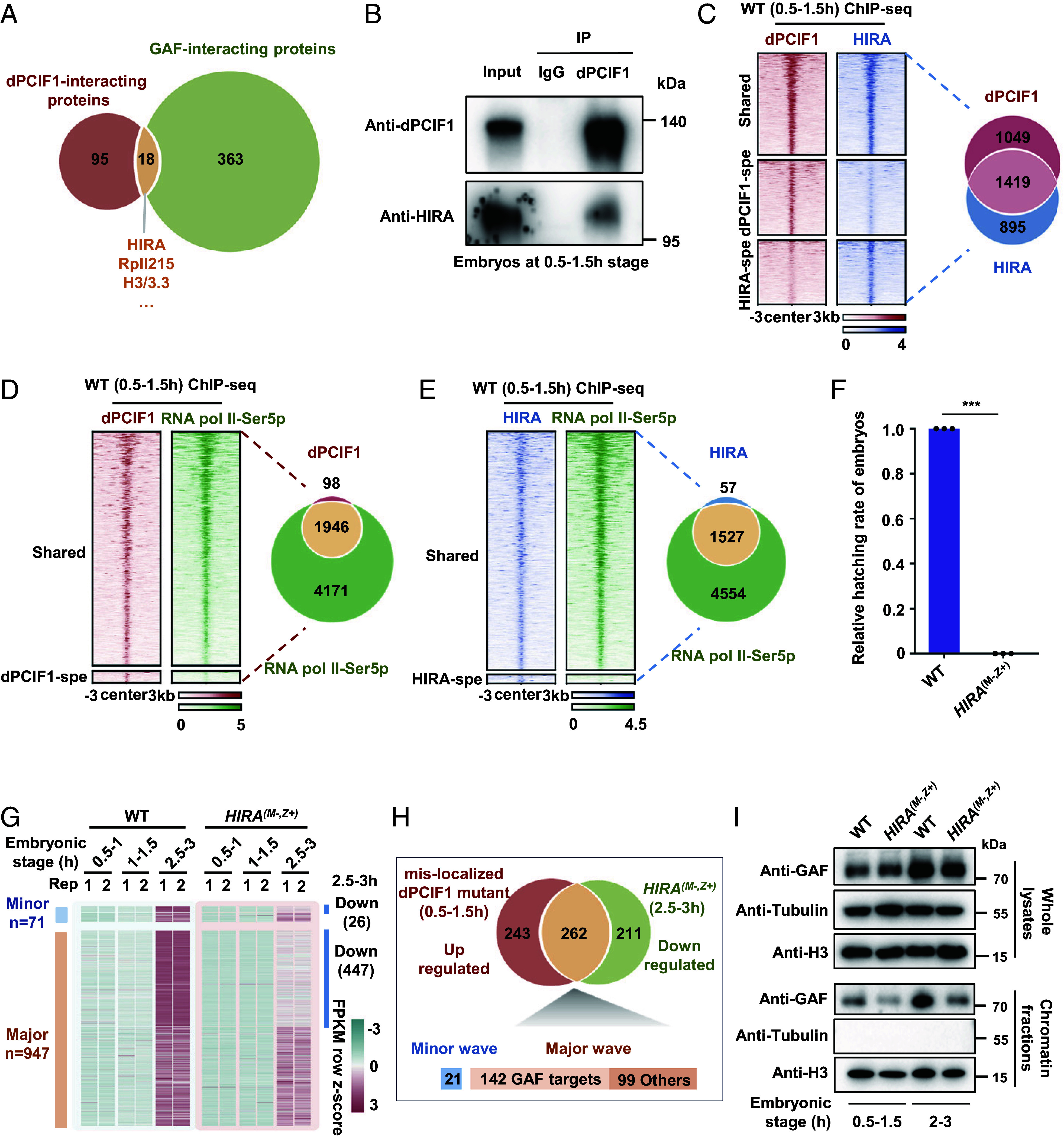
dPCIF1 binds HIRA to restrict GAF function to maintain proper ZGA. (*A*) Venn diagram showing overlapping of proteins interacted with GAF and dPCIF1. (*B*) Co-IP showing the interaction of endogenous dPCIF1 with HIRA in wild-type embryos. (*C*) Heatmap showing dPCIF1 and HIRA occupancy in wild-type embryos. Shared, regions that are bound by both dPCIF1 and HIRA. dPCIF1-spe, regions that are specially bound by dPCIF1. HIRA-spe, regions that are specially bound by HIRA. (*D*) Heatmap showing dPCIF1 and RNA Pol II-Ser5p occupancy at promoter regions in wild-type embryos. Shared, regions that are bound by both dPCIF1 and RNA Pol II-Ser5p. (*E*) Heatmap showing HIRA and RNA Pol II-Ser5p occupancy at promoter regions in wild-type embryos. Shared, regions that are bound by both HIRA and RNA Pol II-Ser5p. (*F*) Bar plot showing the relative hatching rate of *HIRA* maternal mutant embryos. Error bars indicate mean ± SD (n = 3). ***P < 0.001. (*G*) Heatmap showing expression of minor and major wave zygotic transcripts in wild-type and *HIRA* maternal mutant embryos. (*H*) Venn diagram showing overlapping of up-regulated genes in mislocalized dPCIF1 mutant embryos and down-regulated genes in *HIRA* maternal mutant embryos. (*I*) Western blot showing the abundance of GAF in whole lysates and chromatin fractions extracted from wild-type and *HIRA* maternal mutant embryos.

In early embryos, ZGA occurs in two phases: the minor wave of ZGA occurs at NC8–NC13, in which nearly a hundred genes are expressed, whereas at NC14, a few thousand genes (major-wave zygotic genes) become activated (3, 36) (*SI Appendix*, Fig. S2*C*). To determine whether and how the loss of maternal dPCIF1 affects ZGA, we collected wild-type and mislocalized dPCIF1 mutant embryos at various early stages and generated multiple corresponding RNA-seq datasets. RNA-seq analysis revealed that depleting dPCIF1 led to premature expression of zygotic genes (*SI Appendix*, Fig. S2 *D*–*F*). Of note, loss of maternal dPCIF1 did not apparently affect levels of maternal mRNAs at the 0.5 to 1 h stage, although it caused a minor change of maternal mRNAs at the later stages (*SI Appendix*, Fig. S2 *G* and *H*). By focusing on embryos at the minor-wave ZGA stage, we identified 458 major-wave zygotic genes that were ectopically expressed (Fig. 2*H*). Gene Ontology (GO) analysis suggested that these ectopically expressed major-wave zygotic genes were highly enriched with the genes that regulate development involved in cell morphogenesis and differentiation (*SI Appendix*, Fig. S2*I*). Intriguingly, by analyzing published datasets (13), we found that a large portion (~52.2%) of these ectopically expressed zygotic genes were targets of the pioneer factor GAF, including the well-known GAF targets, *Ubx*, *Abd-A*, *Abd-B*, *twi,* and *kni* (Fig. 1*H* and *SI Appendix*, Fig. S2*D*). Of note, only ~8.1% of these genes were unique targets of Zelda (Fig. 1*H*). These results suggest that dPCIF1 maintains proper ZGA, at least in part, by primarily antagonizing the GAF function. To test this, we performed chromatin fractionation experiments. In wild-type embryos, chromatin-associated GAF displayed a dynamic pattern: low levels at the 0.5 to 1.5 h stage but relatively high levels at the 2 to 3 h stage (Fig. 1*I*). However, in *dPCIF1* maternal mutant embryos, the levels of GAF associated with chromatin were markedly increased at the 0.5 to 1.5 h stage compared with the wild-type control (Fig. 1*I*), suggesting a role of dPCIF1 in restricting GAF to efficiently access chromatin at the stage before major-wave gene activation. Of note, no difference in total GAF levels between the wild-type and mutant was found in early embryos (Fig. 1*I*). Intriguingly, co-IP and in vitro pull-down assays revealed no apparent association between dPCIF1 and GAF (Fig. 1*J* and *SI Appendix*, Fig. S2*J*), suggesting an uncharacterized mechanism by which dPCIF1 restricts the GAF function at the early embryonic stage.

### dPCIF1 Maintains Proper ZGA by Binding HIRA to Restrict the GAF Function.

We reasoned that regulation of dPCIF1 restricting the GAF function likely involves additional chromatin-associated factors. To search for the potential factor(s), we performed co-IP assays of embryos at the 2 to 3 h stage, followed by mass spectroscopic analysis, and generated a dataset for GAF-interacting proteins (*SI Appendix*, Fig. S3*A*). By overlapping this dataset with the dPCIF1-interacting protein dataset, we identified a number of protein candidates that potentially interacted with both dPCIF1 and GAF (Fig. 2*A*). Of particular interest, we found that the H3.3-specific chaperone HIRA is the most significantly overlapped factor. In addition, H3/3.3 and RpII215 (Pol II subunit) were also detected to be associated with both dPCIF1 and GAF (Fig. 2*A*). GAF has been reported to control H3.3 replacement by acting in conjunction with HIRA at chromatin boundaries to inhibit the spread of silent chromatin (37). Because dPCIF1 and HIRA form a complex in early embryos, as validated by co-IP analysis (Fig. 2*B*), we explored the regulatory relationship between dPCIF1 and HIRA at the early embryonic stage. We collected embryos at the 0.5 to 1.5 h stage and used anti-dPCIF1 and anti-HIRA antibodies to perform chromatin-immunoprecipitation assays, followed by high-throughput sequencing analysis (ChIP-seq). By analyzing the ChIP-seq datasets between dPCIF1 and HIRA, we found that peaks of dPCIF1 and HIRA were enriched at promoters, and ~61% of HIRA peaks overlapped with dPCIF1 peaks (Fig. 2*C* and *SI Appendix*, Fig. S3 *B* and *C*). Because dPCIF1 is a RNA Pol II-Ser5p associated factor (*SI Appendix*, Fig. S3*D*), we generated ChIP-seq datasets for RNA Pol II-Ser5p at early embryonic stages. Notably, at promoter regions, ~95% of dPCIF1 peaks and ~96% of HIRA peaks overlapped with RNA Pol II-Ser5p peaks (Fig. 2 *D* and *E* and *SI Appendix*, Fig. S3*B*). These findings suggest that dPCIF1 and HIRA share common zygotic targets during ZGA.

Next, we used the CRISPR/Cas9 method to produce two null mutant alleles of HIRA and generated *HIRA* maternal mutant embryos (*SI Appendix*, Fig. S3 *E* and *F*). The hatching rate analysis revealed that loss of HIRA led to complete embryonic lethality (Fig. 2*F*). As shown in *SI Appendix*, Fig. S3*G*, at the 2 to 3 h stage, ~25% of mutant embryos reached NC14, and ~75% of embryos had arrested or delayed nuclear divisions, including a portion of mutant embryos likely displaying no nuclear division. We next used *HIRA* maternal mutant embryos to generate RNA-seq datasets at the various stages and found that loss of maternal HIRA caused global downregulation of zygotic genes, including both minor- and major-wave zygotic genes (Fig. 2*G*). Of note, levels of the maternal stable mRNAs were not significantly reduced in *HIRA* maternal mutant embryos when compared to wild-type control (*SI Appendix*, Fig. S3*H*). Interestingly, by analyzing the RNA-seq datasets between HIRA and dPCIF1, we found that ~51.9% of up-regulated zygotic genes in the dPCIF1 mislocalized mutant (0.5 to 1.5 h) were overlapped with the down-regulated zygotic genes in *HIRA* maternal mutant embryos (2 to 3 h) (Fig. 2*H*). By analyzing these overlapped genes between the two mutants, we found that nearly 92% (241/262) of these overlapped genes were major-wave genes (Fig. 2*H*). Intriguingly, nearly 59% of these overlapped major-wave genes were potential targets of GAF (Fig. 2*H*). Moreover, by performing chromatin fractionation experiments, followed by western blotting, we found that maternal loss of HIRA greatly reduced GAF levels in the chromatin fraction (Fig. 2*I*). These findings suggest that dPCIF1 acts as a surveillance factor, at least in part, to fine-tune the overactivated function of GAF by targeting HIRA.

### HIRA Contributes to Establishing a Totipotent State for Pioneer Factors to Access Chromatin and Generate Efficient Accessible Domain.

**Fig. 3.**
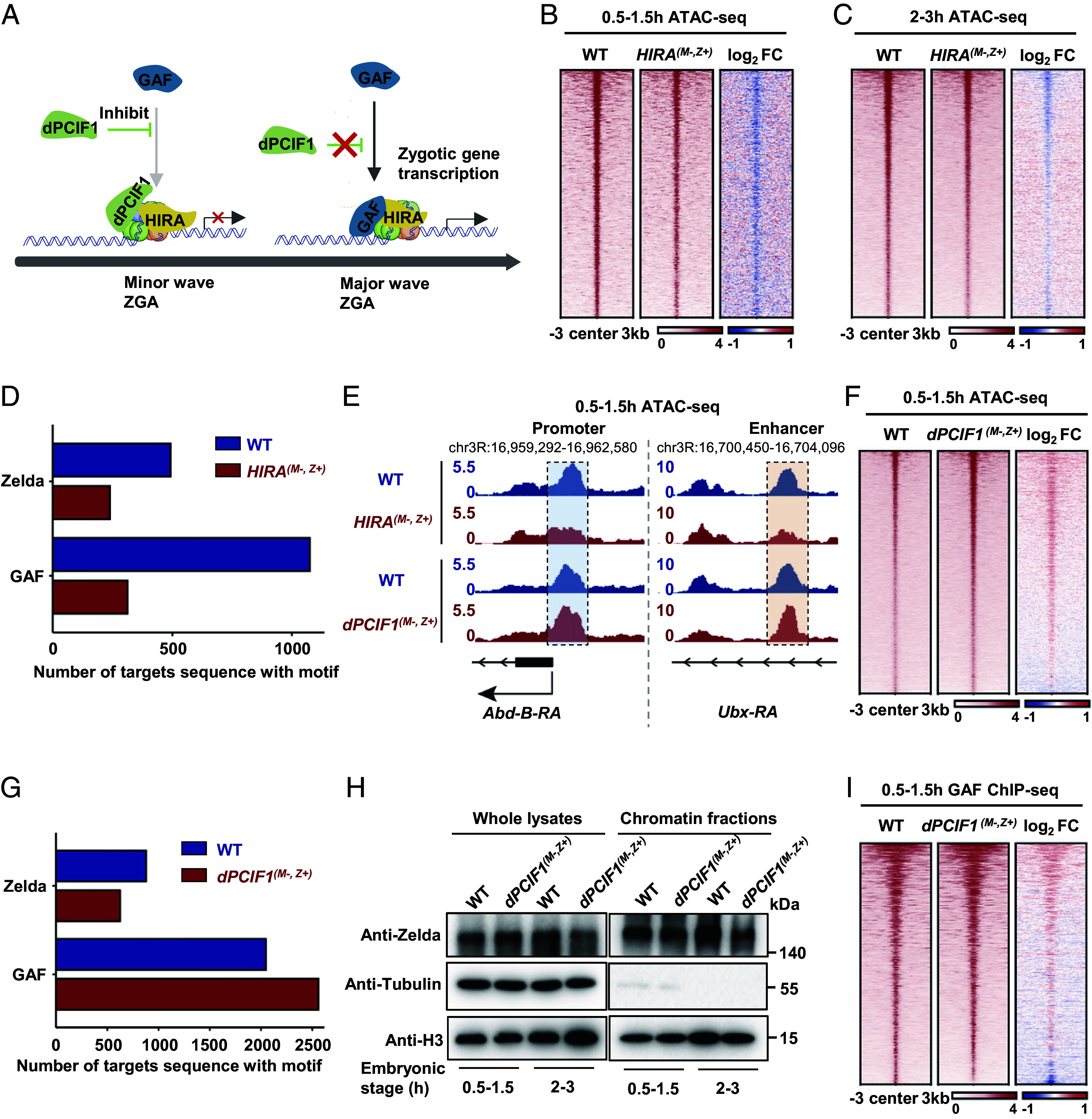
HIRA is critical for pioneer factors to access chromatin. (*A*) Schematic representation illustrating a hypothesized model in which HIRA acts as an upstream factor of GAF. (*B* and *C*) Heatmap showing accessible chromatin signals in wild-type and *HIRA* maternal mutant embryos. (*D*) Bar plot showing the number of accessible chromatin peaks which contained Zelda- and GAF-binding motifs in the 0.5- to 1.5-h embryos with indicated genotypes. (*E*) Integrative genomic viewer (IGV) diagram displaying ATAC-seq signals on selected genes in the 0.5 to 1.5 h embryos with indicated genotypes. (*F*) Heatmap showing accessible chromatin signals in wild-type and *dPCIF1* maternal mutant embryos. (*G*) Bar plot showing the number of accessible chromatin peaks which contained Zelda- and GAF-binding motifs in the 0.5 to 1.5 h embryos with indicated genotypes. (*H*) Western blot showing the abundance of Zelda in whole lysates and chromatin fractions extracted from wild-type and *dPCIF1* maternal mutant embryos at two different stages. (*I*) Heatmap showing GAF occupancy in wild-type and *dPCIF1* maternal mutant embryos.

Based on the above findings, we hypothesized that HIRA may be an upstream factor of GAF. Given that HIRA functions as the H3.3-specific chaperone involved in nucleosome assembly, we speculated that HIRA plays a role in establishing a totipotent state of zygotic chromatin for GAF to bind to nucleosomes, while as an important complement, dPCIF1 restricts GAF activity at the earlier stage, allowing orderly ZGA to occur (Fig. 3*A*). To test this hypothesis, we next investigated accessible chromatin in wild-type and *HIRA* maternal mutant embryos at the 0.5 to 1.5 and 2 to 3 h stages by performing transposase-accessible chromatin with high-throughput sequencing (ATAC-seq) assays (38). We generated two replicate datasets from ATAC-seq of samples at each stage. We identified 3115 and 7569 ATAC-seq peaks at the 0.5 to 1.5 and 2 to 3 h stages of wild-type embryos, respectively, and more than 70% of ATAC-seq signals were enriched in promoter and enhancer regions (*SI Appendix*, Fig. S4*A*). Peaks identified in ATAC-seq were highly consistent with previous findings (*SI Appendix*, Fig. S4*B*) (39). GAF- and Zelda-binding motifs represented the two largest proportions of accessible domains in early chromatin (*SI Appendix*, Fig. S4*C*). Intriguingly, less enrichment of ATAC-seq signals was captured in *HIRA* maternal mutant embryos compared with wild-type embryos (Fig. 3 *B* and *C*). In particular, the largest proportion of these reduced ATAC-seq signals in the *HIRA* mutant was correlated to GAF (Fig. 3*D*). For example, ATAC-seq signals in the promoter or enhancer regions of GAF targets were markedly reduced (Fig. 3*E* and *SI Appendix*, Fig. S4*D*). Additionally, Zelda-related ATAC-seq signals were also reduced (Fig. 3*D*). These findings support HIRA playing a role in establishing the totipotent state for GAF to efficiently access chromatin and generate accessible domain.

Considering that HIRA and dPCIF1 have opposing roles in ZGA, we further generated the ATAC-seq datasets from *dPCIF1* maternal mutant embryos and found that the levels of ATAC-seq signals at the promoter were significantly increased in *dPCIF1* maternal mutant embryos at the very early stage compared with the wild-type control (Fig. 3*F*). Because loss of maternal dPCIF1 causes premature activation of zygotic genes, many of which are GAF targets, we determined whether loss of maternal dPCIF1 affects the enrichment of GAF- and Zelda-binding motifs in early chromatin. As shown in Fig. 3*G*, we observed a relatively weak reduction in signals of the putative Zelda-binding motif in ATAC-seq peaks at promoter regions between wild-type and *dPCIF1* maternal mutant embryos. However, signals of putative GAF-binding motifs in ATAC-seq peaks at promoter regions were markedly increased in *dPCIF1* maternal mutant embryos compared to wild-type embryos (Fig. 3*G*). For example, ATAC-seq signals in the promoter or enhancer regions of GAF targets, such as *Ubx* and *Abd-B*, were apparently increased (Fig. 3*E* and *SI Appendix*, Fig. S4*D*). In support of this, biochemical assays suggested that the GAF level was significantly increased in the chromatin fraction of *dPCIF1* maternal mutant embryos compared with that in wild-type embryos (Fig. 1*I*). Conversely, no apparent change in the levels of Zelda associated with early chromatin was found between wild-type and *dPCIF1* maternal mutant embryos (Fig. 3*H*). Next, we generated ChIP-seq datasets for GAF and Zelda from wild-type and *dPCIF1* maternal mutant embryos at the early stage. More than 70% of ChIP-seq signals for GAF and Zelda were enriched in promoter and enhancer regions (*SI Appendix*, Fig. S4*E*). It is worth to note that GAF ChIP peaks were highly enriched in the sequences that contained GAF motifs (*SI Appendix*, Fig. S4*F*). These results were consistent with a previous study (*SI Appendix*, Fig. S4*G*) (13). Similar results were obtained when Zelda ChIP datasets were analyzed (*SI Appendix*, Fig. S4*H*). Further Zelda peaks showed no increased ChIP-seq signals (*SI Appendix*, Fig. S4*I*), whereas ChIP-seq signals for GAF peaks were markedly increased in *dPCIF1* maternal mutant embryos at the early stage compared with the wild type (Fig. 3*I*). Additionally, we generated GAF-ChIP-seq datasets from wild-type and *HIRA* maternal mutant embryos at the 2- to 3-h stage and found that the loss of HIRA markedly reduced the accessibility of GAF on early chromatin (*SI Appendix*, Fig. S4*J*). These findings support that dPCIF1 coordinates with HIRA to permit GAF to properly access chromatin at the major-wave ZGA stage.

### HIRA and H3.3 Coordinate to Establish the Totipotent State of Early Chromatin.

**Fig. 4.**
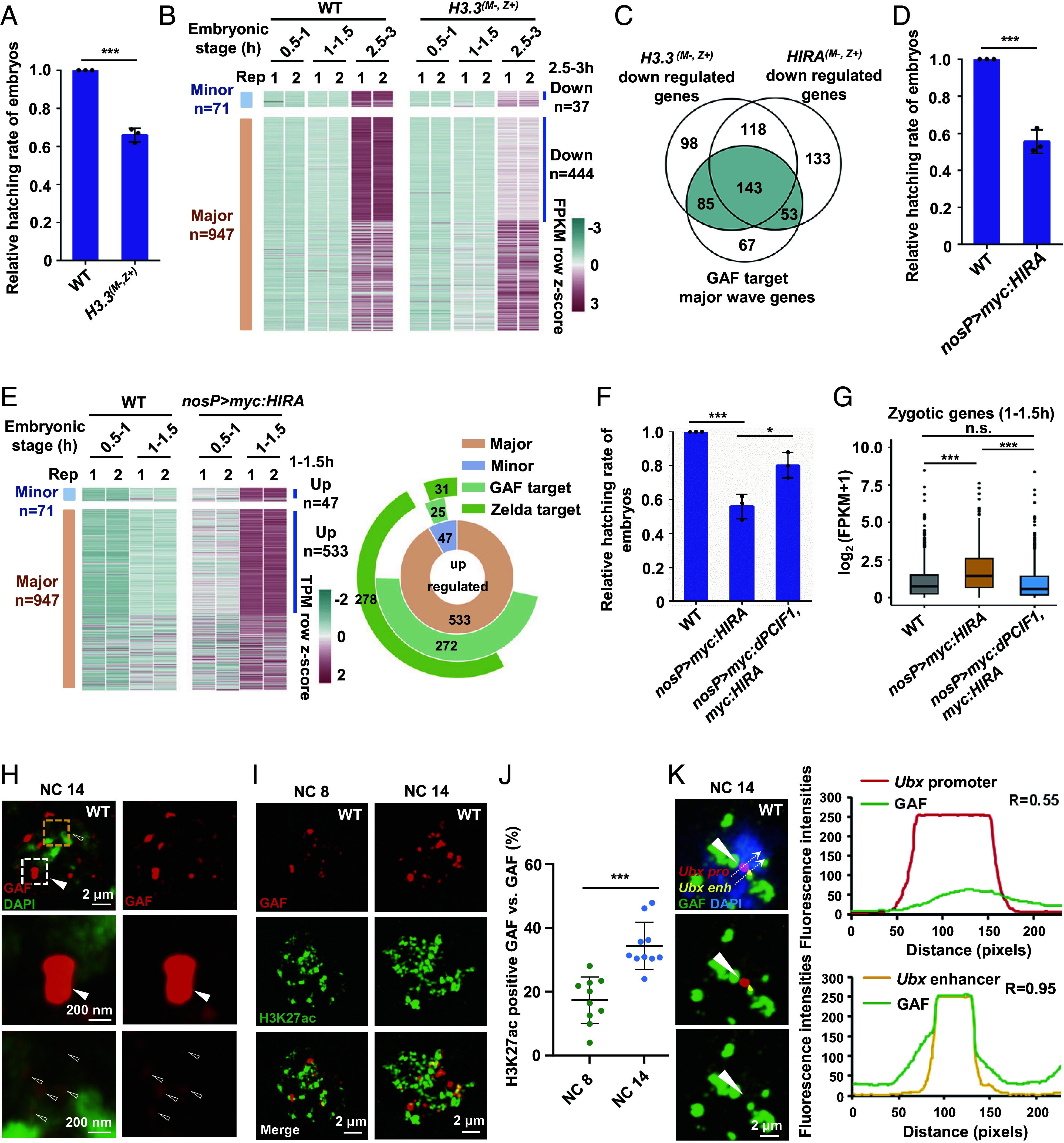
HIRA and H3.3 coordinate to establish the totipotent state of early chromatin. (*A*) Bar plot showing the relative hatching rate of *H3.3* maternal mutant embryos. (*B*) Heatmap showing expression of minor and major wave zygotic genes in wild-type and *H3.3* maternal mutant embryos at three different stages. (*C*) Venn diagram showing overlapping of three gene categories. (*D*) Bar plot showing the relative hatching rate of maternal overexpressed HIRA embryos. (*E*) *Left* panel, heatmap showing expression of minor and major wave zygotic transcripts in wild-type and maternal overexpressed HIRA embryos at two different stages. *Right* panel, Venn pie chart displaying the overlapping of four gene groups. (*F*) Bar plot showing the relative hatching rate of embryos with indicated genotypes. (*G*) Box plot showing the expression levels of zygotic genes in indicated samples. (*H*) Immunostaining showing that GAF can form two types of cellular granules, large-sized granules (indicated by the white dotted box) with strong GAF signal and small-sized granules (indicated by the yellow dotted box). (Scale bars, as indicated.) (*I*) Immunostaining showing colocalization of GAF and H3K27ac signals in wild-type embryos at indicated stages. (Scale bars, 2 μm.) (*J*) Scatter plot showing that the percentage of GAF granules were positive for H3K27ac in (*I*). (*K*) Immunostaining combined with DNA-FISH showing that *Ubx* enhancer and promoter were simultaneously overlapped with GAF signals. The *Right* panel shows the Pearson’s correlation coefficients of GAF and the *Ubx* regions. Error bars indicate mean ± SD (n = 3). *P < 0.05, ***P < 0.001, n.s., not significant.

Considering that HIRA plays a role in assembling histone H3.3-containing nucleosomes, we examined whether and how the H3.3 variant affects ZGA in early embryos. The *Drosophila* genome harbors two genes encoding H3.3, namely *H3.3A* and *H3.3B*. To study the function of H3.3, we generated multiple *H3.3A* and *H3.3B* mutant alleles by the CRISPR/Cas9 method (*SI Appendix*, Fig. S5*A*). Consistent with previous findings (40), we found that although *H3.3A* and *H3.3B* double mutants displayed dramatically reduced viability, they yielded a small fraction of adult “escapers.” While these adult homozygous mutant males were sterile, the homozygous mutant females could produce normal number of eggs. By taking this advantage, we generated *H3.3A* and *H3.3B* maternal double-mutant embryos and then carried out embryo hatching rate analysis. As shown in Fig. 4*A*, loss of maternal H3.3 caused a strong embryonic lethality because ~40% of mutant embryos failed to reach the larval stage, suggesting that the histone H3.3 variant is important for embryonic development. To determine whether H3.3 affects ZGA similarly to HIRA, we collected *H3.3* maternal mutant embryos at early stages and generated RNA-seq datasets. RNA-seq analysis showed that loss of maternal H3.3 resulted in a global reduction of zygotic gene expression, including both minor- and major-wave zygotic genes (Fig. 4*B*). Interestingly, nearly 60% of down-regulated major-wave zygotic genes in *H3.3* maternal mutant embryos overlapped with those in *HIRA* maternal mutant embryos (Fig. 4*C*). Of note, the largest proportion (~55%) of these overlapped genes were GAF targets (Fig. 4*C*). These findings emphasize that the incorporation of H3.3 into the nucleosome by HIRA plays an important role in contributing to the induction of ZGA. Because a large proportion of HIRA and H3.3 targets significantly overlapped with dPCIF1 targets (*SI Appendix*, Fig. S5*B*), we individually overexpressed maternal HIRA or H3.3 in early embryos. Hatching rate analysis revealed that overexpression of HIRA, but not H3.3, caused a strong embryonic lethal phenotype (Fig. 4*D* and *SI Appendix*, Fig. S5 *C* and *D*). Immunostaining assays indicated that HIRA overexpression caused premature zygotic gene activation because RNA Pol II-Ser5p and H3K27ac signals were detected even early at NC5 and NC7, respectively (*SI Appendix*, Fig. S5 *E* and *F*). ChIP-seq analysis revealed that HIRA overexpression increased the accessibility of GAF at the early stage (*SI Appendix*, Fig. S5*G*). RNA-seq analysis revealed that HIRA overexpression up-regulated 580 zygotic genes, including 47 minor-wave genes and 533 major-wave genes, at the 1 to 1.5 h stage (Fig. 4*E*). Among them, while a few genes were Zelda targets, 297 genes were GAF targets. These findings emphasize the critical role of HIRA in coordination with pioneer factors to regulate ZGA. To examine whether the phenotype induced by HIRA overexpression was partly attributed to bypassing endogenous dPCIF1 activity, we co-overexpressed dPCIF1 with HIRA in early embryos and found that overexpressed dPCIF1 significantly suppressed the embryonic lethal phenotype induced by HIRA overexpression (Fig. 4*F* and *SI Appendix*, Fig. S5*H*). Importantly, by analyzing RNA-seq datasets, we found that co-overexpression of dPCIF1 with HIRA recovered the expression of 78% (454/580) of up-regulated genes to the levels observed in wild-type embryos at the 1 to 1.5 h stage (Fig. 4*G* and *SI Appendix*, Fig. S5*I*). Among them, 243 genes were GAF targets. Collectively, these results support that dPCIF1 functions as a surveillance factor to facilitate HIRA establishing orderly ZGA by restricting premature activation of GAF.

### Nucleosome Binding Promotes Condensation of GAF that Contributes to ZGA.

As a pioneer factor, GAF harbors a BTB domain, a DNA-binding domain (DBD) domain, and an intrinsically disordered low complexity sequence (LC) at its C-terminal region, including a polyQ sequence (*SI Appendix*, Fig. S6*A*) (41, 42). Consistent with the findings from previous studies (43, 44), our co-IP analysis showed that the BTB domain, but not the DBD domain or the polyQ domain, is important for self-interaction of GAF (*SI Appendix*, Fig. S6*B*). Similar to many transcription factors, pioneer factors bind directly to DNA through their DBD domain. However, DBDs in some pioneer factors also bind to nucleosomes with high affinity (45–47). As shown by co-IP assays, GAF was associated with histone proteins with relatively high affinity through its DBD (*SI Appendix*, Fig. S6 *C*–*F*), indicating that GAF binds to nucleosomes. To search for evidence that GAF directly interacts with nucleosomes, we purified bacterially expressed H2A, H2B, H3 (or H3.3), and H4 proteins and used these histone proteins together with double-stranded DNA containing the GAF-binding motif to prepare two types of nucleosome core particles containing H3 (NCP(H3)) or containing H3.3 (NCP(H3.3)) (*SI Appendix*, Fig. S7 *A*–*C*). In vitro pull-down assays showed that GST-GAF, but not GST-GAF^del-DBD^ (a mutant form of GAF lacking its DBD), directly pulled down nucleosomes (*SI Appendix*, Fig. S7*D*), suggesting that GAF binds to nucleosomes directly through its DBD. Next, we determined whether binding to nucleosomes affects the behavior of GAF and thus performed in vitro phase separation assays. As shown in *SI Appendix*, Fig. S7*E*, GAF alone or GAF in combination with free histone proteins were evenly distributed in solution at a physiological salt concentration (150 mM NaCl). However, in the presence of nucleosomes, GAF unexpectedly began to condense with nucleosomes to form protein droplets (2.4 µm diameter) (*SI Appendix*, Fig. S7*F*). Of note, deletion of BTB or DBD markedly affected the cophase separation of GAF with nucleosomes (*SI Appendix*, Fig. S7 *F* and *G*). By carrying out semidenaturing detergent agarose gel electrophoresis analysis (48), which detects prion-like protein aggregates, we found that the strongest oligomer signals of GAF were detected in solution in the presence of nucleosomes (*SI Appendix*, Fig. S7*H*), suggesting that nucleosomes promote GAF to condensate into high molecular weight oligomers. Consistent with its phase separation behavior, GAF lacking BTB or DBD domain markedly reduced the formation of high molecular weight oligomers (*SI Appendix*, Fig. S7*I*). Next, we used anti-GAF antibody to carry out immunostaining assays in wild-type embryos at early stages using a confocal microscope with Airyscan 2 superresolution. As shown in Fig. 4*H*, we found that GAF formed two types of cellular granules: large-sized granules (200 to 400 nm diameter) with strong GAF signal and small-sized granules (~100 nm diameter) with relatively weak GAF signal in early embryos. A previous study reported that GAF granules are involved in establishing heterochromatin during development (41). Consistent with this, immunostaining combined with DNA-FISH assays showed that most large-sized granules were associated with the (AAGAG)_7_ repeats signal (*SI Appendix*, Fig. S7*J*). Because GAF is essential for ZGA, we determined whether GAF granules are also associated with active zygotic genes. We performed further immunostaining assays to examine whether GAF granules overlapped with an active histone marker using anti-GAF and anti-H3K27ac antibodies. As shown in Fig. 4 *I* and *J*, at the early (1 to 1.5 h) embryonic stage, only a very small proportion of GAF granules harbored H3K27ac-positive signals, but at the later (2 to 3 h) stage, a large portion of small-sized GAF granules and some large-sized granules showed H3K27ac-positive signals, indicating that at least, a subtype of GAF granules associate with active zygotic genes. Next, we analyzed dPCIF1 ChIP-seq datasets and GAF ChIP-seq datasets from wild-type and *dPCIF1* maternal mutant embryos and identified 272 GAF-regulated zygotic genes that were also targeted by dPCIF1 (*SI Appendix*, Fig. S7*K*). These genes included well-known target genes of GAF, such as *Ubx* and *Abd-B*. To determine whether GAF granules associate with target genes at promoter regions at NC14, we performed DNA-FISH combined with immunostaining assays. As shown in *SI Appendix*, Fig. S7*L*, GAF granules could be overlapped with FISH signals targeting the promoter regions of *Ubx* and *Abd-B* genes. Moreover, as expected, the enhancer and promoter of *Ubx* could be simultaneously overlapped with GAF signals (Fig. 4*K*). These findings provide a direct line of evidence showing that GAF granules can associate with active zygotic genes.

### HIRA and dPCIF1 Coordinately Determine the Phase Separation Behavior and Function of GAF in Early Embryos.

**Fig. 5.**
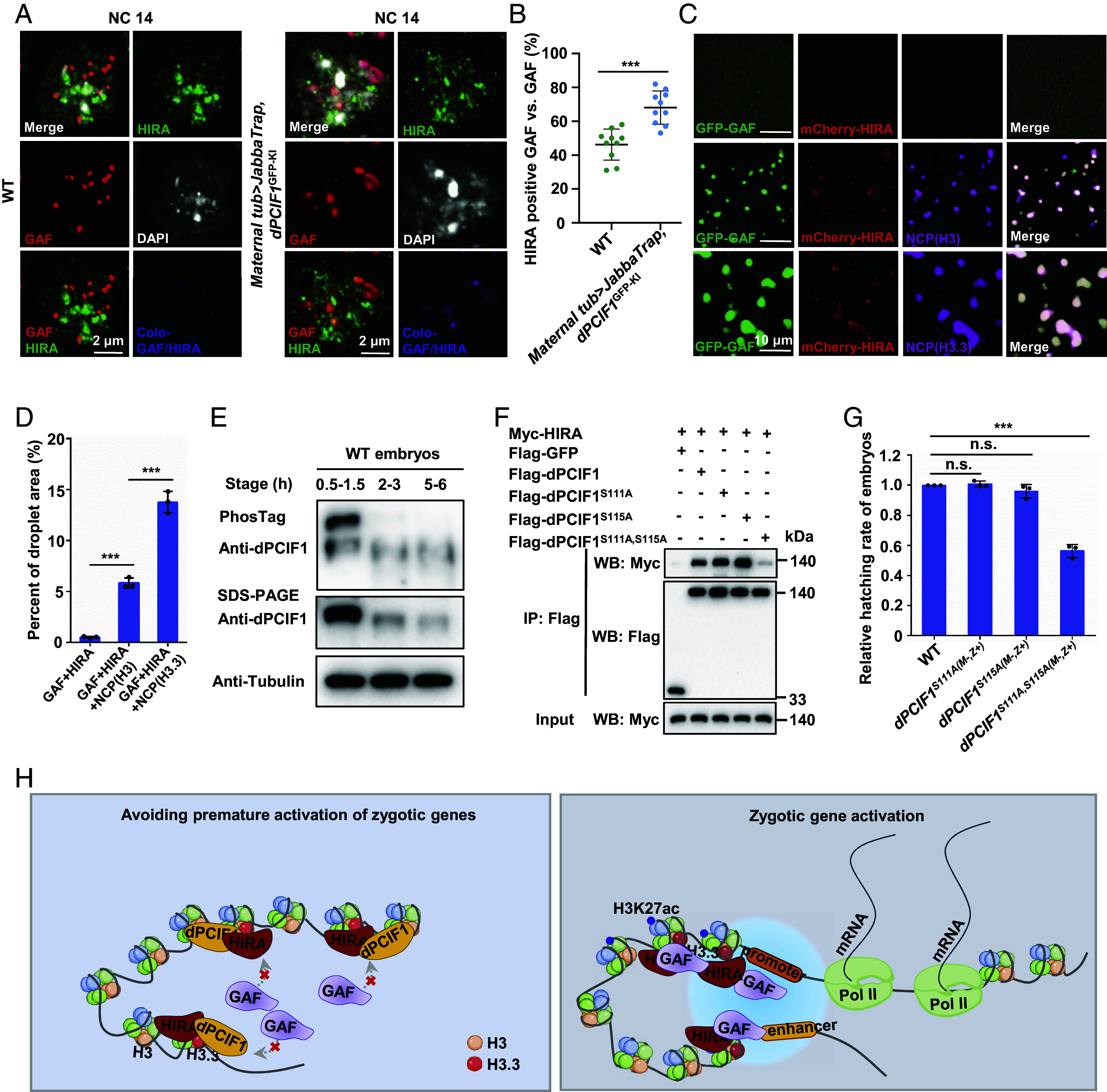
HIRA and dPCIF1 coordinately determine the phase separation and function of GAF in early embryos. (*A*) Immunostaining showing the colocalization of GAF and HIRA in wild-type and mislocalized dPCIF1 mutant embryos. (Scale bars, 2 μm.) (*B*) Scatter plot showing that the percentage of GAF granules were positive for HIRA in (*A*). (*C*) Droplet formation assays for GFP-GAF (1 mg/mL) mixed with mCherry-HIRA (1 mg/mL) and NCP(H3 or H3.3) (0.1 μM). (Scale bars, 10 μm.) (*D*) The quantitative area of green droplet signal in (*C*). (*E*) Phos-tag gel electrophoresis assay showing the phosphorylated dPCIF1 proteins. (*F*) Co-IP showing the interaction of Myc-HIRA with Flag-tagged wild-type and mutant dPCIF1 in S2 cells. (*G*) Bar plot showing the relative hatching rate of embryos with indicated genotypes. (*H*) Model for dPCIF1 competitively binds to HIRA to antagonize the GAF–HIRA function, allowing orderly ZGA. Error bars indicate mean ± SD (n = 3). ***P < 0.001, n.s., not significant.

Because GAF activates major-wave zygotic gene expression by interaction with HIRA, which is restricted by dPCIF1 at the early stage, we next explored the biochemical and biophysical behaviors of these three proteins on early chromatin. Double immunostaining assays using anti-GAF and anti-HIRA antibodies showed that ~46% of GAF granules were positive for HIRA (Fig. 5 *A* and *B*), as calculated by IMARIS9.0.1 software. However, in mislocalized dPCIF1 mutant embryos, ~68% of GAF granules were positive for HIRA, and the size of these GAF granules was greatly increased to 400 to 700 nm in diameter (Fig. 5 *A* and *B*). These findings suggest that the behavior of GAF granules is highly dynamic and likely regulated by dPCIF1 in early embryos.

To understand the biophysical properties of GAF granules regulated by HIRA and dPCIF1, we carried out further in vitro phase separation assays. As mentioned above, GAF and NCP(H3) or NCP(H3.3) form phase-separated droplets (average 2 µm diameter) (*SI Appendix*, Fig. S8 *A* and *B*). Interestingly, in the presence of HIRA protein together with NCP(H3.3), but not NCP(H3), GAF formed larger droplets (average 5 µm diameter) (Fig. 5 *C* and *D*), suggesting that HIRA promotes phase separation of GAF with NCP(H3.3), but not NCP(H3). To examine how HIRA affects the phase separation of GAF with NCP(H3.3), we performed domain-mapping experiments by generating numerous deletion mutants of HIRA and GAF. As shown by co-IP assays, the BTB domain was essential for GAF to associate with HIRA, while the N-terminal region (1 to 400 aa), HIRA^N^, was critical for HIRA to associate with GAF (*SI Appendix*, Fig. S8 *C*–*E*). Interestingly, deletions of the BTB domain in GAF or deletion of the N-terminal region in HIRA appeared to suppress the phase separation process (*SI Appendix*, Fig. S8 *F*–*I*), suggesting that the interaction between HIRA and GAF is essential to drive their cophase separation. Moreover, deletion of the DBD in GAF completely abolished its phase separation with NCP(H3.3), even in the presence of HIRA (*SI Appendix*, Fig. S8 *F* and *G*). Of note, deletion of polyQ did not apparently affect GAF cophase separation with HIRA and NCP(H3.3), suggesting that this intrinsically disordered LC region is not a driver for phase separation in the context of GAF-HIRA-NCP(H3.3). To investigate whether dPCIF1 affects HIRA-mediated phase separation of GAF-NCP(H3.3), we mixed dPCIF1, GAF, HIRA, and NCP(H3.3) together in solution and found that addition of dPCIF1 significantly inhibited HIRA-mediated phase separation of GAF-NCP(H3.3) (*SI Appendix*, Fig. S8 *J* and *K*).

We next reanalyzed the pattern of the dPCIF1 protein at various stages and found that dPCIF1 had a larger band at the 0.5 to 1.5 h stage than that at the later stage (Fig. 1*A*). To examine whether dPCIF1 was phosphorylated, we performed Phos-tag gel electrophoresis to separate potentially phosphorylated forms of dPCIF1. As shown in Fig. 5*E* and *SI Appendix*, Fig. S8*L*, dPCIF1 was indeed phosphorylated at the early stage because lambda phosphatase treatment abolished the shift band. To identify the site(s) of dPCIF1 phosphorylation, we used the 0.5 to 01.5 h embryos to perform phosphor-proteomics by mass spectroscopy and identified the Ser-111 as a phosphorylation site and the Ser-115 as a potential phosphorylation site (*SI Appendix*, Fig. S8*M*). To determine whether dPCIF1 phosphorylation affects its interaction with HIRA, we generated several mutant forms of dPCIF1, dPCIF1^S111A^, dPCIF1^S115A^, and dPCIF1^S111A, S115A^, which mimicked unphosphorylated forms of dPCIF1 (*SI Appendix*, Fig. S8*N*), respectively. Co-IP showed that double mutation of S111 and S115 to A, but not single mutation of S111 or S115 to A, markedly reduced the HIRA–dPCIF1 interaction (Fig. 5*F*), suggesting that phosphorylation is important for dPCIF1 to target HIRA. To assess the biological function of dPCIF1 phosphorylation, we mutated S111 and/or S115 sites to A by generating three knock-in strains using the CRISPR/Cas9 technique. By generating maternal mutant embryos, we found that mutation of the two sites, but not the single site, led to embryonic lethality (Fig. 5*G*). Further RNA-seq analysis revealed that mutation of the S111 and S115 sites to A led to premature activation of major-wave zygotic genes at the minor-wave stage (*SI Appendix*, Fig. S8*O*). These findings emphasize the importance of S111 and S115 sites in dPCIF1 in early embryos.

## Discussion

In *Drosophila*, while the pioneer factor Zelda is primarily responsible for initiating the earliest zygotic gene activation, GAF plays a crucial role in driving widespread zygotic transcription, thereby ensuring correct ZGA and successful embryo development. Despite both maternal Zelda and GAF being highly expressed in the earliest stages, GAF performs its major functions at a relatively later stage, suggesting an uncharacterized surveillance mechanism that prevents GAF from prematurely accessing early chromatin. In this study, we present evidence supporting a working model that explains how the coordination between pioneer factors Zelda and GAF ensures the activation of the zygotic genome at the correct time and within the proper spatial context. According to this model, the GAF accesses chromatin by directly interacting with HIRA to activate major-wave zygotic genes, while dPCIF1 acts as a surveillance factor to prevent premature ZGA by competitively binding to HIRA and restricting GAF to access onto early chromatin (Fig. 5*H*). Our study thus reveals that the H3.3-specific chaperone plays a role in establishing totipotent-state chromatin, enabling orderly ZGA in early embryos.

Early embryogenesis is a highly robust process that requires precise regulation, suggesting that additional factors may assist pioneer factors in binding to their motifs on nucleosomes. In this study, we show that HIRA serves as one such factor in *Drosophila* early embryos, contributing to the establishment of totipotent chromatin. It has been documented that HIRA plays an important role in the formation of the diploid zygote (25), and depletion of maternal HIRA results in embryonic lethality. Therefore, it is critical to investigate whether HIRA also plays a role in ZGA, beyond its established function in diploid zygote formation. Our study provides several lines of evidence supporting the involvement of HIRA in the regulation of ZGA. First, ChIP-seq analysis revealed that the genome-wide distribution of HIRA on early chromatin exhibited a DNA sequence-specific pattern. Second, biochemical results indicated that GAF directly binds to HIRA and H3.3 in a domain-dependent manner, and this interaction facilitates GAF binding to H3.3-containing nucleosomes. Third, maternal loss of HIRA reduced chromatin accessibility for GAF, leading to the inactivation of global zygotic gene expression. Fourth, maternal overexpression of HIRA increased chromatin accessibility for GAF, and enhancing GAF target gene expression at the early stage. Finally, loss of maternal H3.3 leads to an abnormal ZGA pattern with reduced GAF target gene expression and an embryonic lethal phenotype. These findings support that, in addition to its established role in forming the diploid zygote, HIRA promotes GAF’s loading onto chromatin. Recent studies have reported that HIRA plays a role in regulating transcriptional programs in other biological systems (49, 50). Given the conservation of HIRA during evolution, we speculate that HIRA-mediated regulation of ZGA may be shared among metazoans.

Fractionation assays showed that GAF displayed a dynamic pattern on chromatin, with low levels at the early stage but high levels at the late stage in embryos. The dynamics of GAF on chromatin might explain why it acts as a maternal pioneer factor but conducts its function at the major-wave stage. In this study, we also found that dPCIF1 is bound to the N-terminal region of HIRA. This binding appeared to reduce the interaction between GAF and HIRA in a competitive manner, thereby restricting premature activity of GAF at the early stage. Interestingly, maternal dPCIF1 has a short half-life in early embryos, and its rapid degradation at the late stage is undoubtedly important for orderly ZGA. Our data suggest that 111-Ser and 115-Ser sites in dPCIF1 are important for its protein stability, indicating an uncharacterized mechanism responsible for this regulation is critical for normal ZGA. Nevertheless, it would be interesting to address this important issue in the future.

## Materials and Methods

In this study, LC–MS/MS was used to identify interacting protein and map the phosphorylation sites, ATAC-seq was used to investigate accessible chromatin, in vitro phase separation assays was employed to analyze the biophysical properties of GAF granules. Details of the experiments, e.g., embryo preparation, immunoprecipitation and western blot, fractionation of embryo chromatin, library sequencing, and data analysis, are described in *SI Appendix*, *Materials and Methods*.

## Supplementary Material

Appendix 01 (PDF)

## Data Availability

The raw sequencing data reported in this paper have been deposited in NCBI’s Gene Expression Omnibus and are accessible through GEO Series (GSE253765) (51). The mass spectrometry proteomics data generated in this study have been deposited to the ProteomeXchange Consortium via the iProX partner repository with the dataset identifier PXD048659 (52). All other data are included in the manuscript and/or *SI Appendix*.
