# Supplementary material for: HIRA and dPCIF1 coordinately establish totipotent chromatin and control orderly ZGA in Drosophila embryos
Source: Proc Natl Acad Sci U S A. 2024 Nov 14;121(47):e2410261121. doi: 10.1073/pnas.2410261121 (PMC11588057; doi:10.1073/pnas.2410261121)
Supplement: Supplementary file 1 — Appendix 01 (PDF) [file pnas.2410261121.sapp.pdf]

PNAS

Supporting Information for

HIRA and dPCIF1 coordinately establish totipotent chromatin and control orderly ZGA in *Drosophila* embryos

Guoqiang Zhang[a, 1], Yaqi Miao[a, 1], Yuan Song[b, c, 1], Liangliang Wang[a, 1], Yawei Li[a], Yuanxiang Zhu[a], Wenxin Zhang[a], Qinmiao Sun[b, c, d, 2], Dahua Chen[a, e, 2]

[a] Institute of Biomedical Research, Yunnan University, Kunming 650500, China

[b] State Key Laboratory of Membrane Biology, Institute of Zoology, Chinese Academy of Sciences, Beijing 100101, China

[c] School of Life Sciences, University of Chinese Academy of Sciences, Beijing 100049, China

[d] Institute of Stem Cells and Regeneration, Chinese Academy of Sciences, Beijing 100101, China

[e] Southwest United Graduate School, Kunming 650092, China

[1] These authors contributed equally to this work.

[2] Correspondence to:

Dahua Chen, **Email:** chendh@ynu.edu.cn

Qinmiao Sun, **Email:** qinmiaosun@ioz.ac.cn

This PDF file includes:

Supplementary Material and Methods
Figures S1 to S8
Tables S1 to S3
SI References

Supplementary Material and Methods

Drosophila strains

The fly stocks used in this study were maintained under standard culture conditions. The *Drosophila* w^{1118} strain was employed for the collection of wild-type embryos and served as the host for P element-mediated transformations. The subsequent fly strains were generated as follows: (1) Mutant strains: $dPCIF1^{1}$, $dPCIF1^{2}$, $dPCIF1^{S111A}$, $dPCIF1^{S115A}$, $dPCIF1^{S111A,S115A}$, $HIRA^{1}$, $HIRA^{2}$, $H3.3A^{1}$, $H3.3A^{2}$, $H3.3B^{1}$, and $H3.3B^{2}$ mutant alleles were created using the previously described method (1). (2) Transgenic strains: This category includes P{*uasp-myc-dPCIF1*}, P{*uasp-myc-HIRA*}, P{*uasp-gfp-H3.3*}, and P{*uasp-VhhGFP4-jabba-VhhGFP4*}. (3) Knock-in strain: P{$dPCIF1^{GFP\text{-}KI}$} is a knock-in strain where the GFP coding sequence was fused to the C-terminus of dPCIF1. Detailed information about the primers can be found in Table S1. Additionally, these lines were provided by the Bloomington *Drosophila* Stock Center (BDSC): (1) w[1118]; Df(3L)BSC563/TM6C, cu[1] Sb[1] (25721#); (2) w[*]; P{w[+mC]=matalpha4-GAL-VP16}V37 (7063#).

Embryo preparation

Drosophila embryo samples were collected using a previously established method (2). In summary, all flies were grown and lay eggs at 25°C. Well-fed flies were allowed to lay eggs in bottles, with each bottle covered by a petri dish containing agar gel and apple juice. The embryos were meticulously collected and inspected under light microscopes. Older embryos were identified and removed using the light microscope, after which the remaining embryos were cleaned with washing buffer (1xPBS) to prevent contamination. Subsequently, the samples were promptly used for follow-up experiments or frozen in liquid nitrogen and stored at -80°C.

m6Am quantification by UHPLC-MRM-MS/MS

The NEBNext Poly(A) mRNA Magnetic Isolation Module Kit (E7490, New England Biolabs) was used to isolate the polyadenylated RNA from the embryos as described below. Initially, 100 ng of isolated mRNAs were digested using nuclease P1 in a 40 µl buffer containing 10 mM NH_4OAc (pH 5.3) for a duration of 6 hours at 42ºC. Following this, NH_4HCO_3 and alkaline phosphatase were added, and the mixture was incubated for an additional 6 hours at 37ºC. The resulting solution was then diluted with ddH_2O to a final volume of 200 µl. Next, 10 µl of the diluted solution was subjected to LC-MS/MS analysis. Reverse phase ultra-performance liquid chromatography on a C18 column was employed to separate the nucleosides, which were subsequently detected using Waters TQ-XS QQQ triple quadrupole LC-MS in positive electrospray ionization mode. The quantification of nucleosides was based on the nucleoside to base ion mass transitions of 295.8-149.98 (m6Am) and 268-136 (A). To quantify the nucleosides, a standard curve was established using pure nucleoside standards that ran

alongside the samples in the same batch. The ratio of m6Am to A was calculated using the determined concentrations in three independent biological replicates.

Analysis of hatching rate

Embryos were collected over a 24-hour period at 25°C, then separated from the adults and left to develop for an additional 36 hours. Subsequently, the number of unhatched embryos and larvae was tallied to determine the hatching rate. The relative hatching rate of embryos was calculated in three independent biological replicates.

RNA sequencing

Total RNAs were isolated from hand selected embryos (50 embryos per replicate) of the indicated genotypes and stages in two biological replicates using TRIzol reagent. RNA samples (0.5 µg total RNA each) were first rRNA-depleted. The strand-specific libraries were made using VAHTS Universal V8 RNA-seq Library Prep Kit for MGI (NRM605, Vazyme) according to the manufacturer's instructions and sequenced using the MGISEQ-2000RS platform.

Antibody production

The antibodies were generated by immunizing mice or rabbits with recombinant proteins that were produced in *Escherichia coli*. The recombinant proteins were used as follows: MBP-dPCIF1 (Amino acids 1-200 aa), GFP-dPCIF1^{FL}, MBP-GAF (Amino acids 52-202 aa), MBP-HIRA (Amino acids 501-700 aa) and MBP-Zelda (Amino acids 351-721 aa).

Immunoprecipitation and western blot

The embryos or S2 cells transfected with the plasmids were homogenized with lysis buffer (50 mM Tris-HCl, pH 7.4, 1 mM EDTA, 150 mM NaCl, 10% glycerol and 1% NP-40) with protease inhibitors. The supernatant was incubated with specific antibodies overnight at 4°C, and then Pierce™ protein A/G agarose (20422, Thermo Fisher), anti-GFP nanobody agarose (KTSM1301, AlpaLifeBio) or Flag beads (A2220, Sigma) were added and incubated for another 2 hours at 4°C. The beads were washed extensively with lysis buffer three times. The immunoprecipitates were eluted with SDS sample buffer and analyzed by western blot. Rabbit anti-dPCIF1 (1:100), anti-GFP beads and Flag beads were used for immunoprecipitation. Mouse anti-β-Tubulin (1:2000, CW0098M, CWBIO), mouse anti-dPCIF1 (1:1000), mouse anti-HIRA (1:1000), mouse anti-GAF (1:1000), mouse anti-Zelda (1:1000), rabbit anti-H3 (1:2000, ab176842, Abcam), rabbit anti-H4 (1:2000, ab177840, Abcam), rabbit anti-Myc (1:2000, 562, MBL), rabbit DDDDK-tag (1:2000, PM020, MBL), rabbit anti-RNA polymerase II-phospho S5 (1:5000, ab5131, Abcam) and rabbit anti-GFP (1:3000) were used for western blot. The

quantitation of band intensity was measured using ImageJ software from three biological replicates.

Fractionation of embryo chromatin

60 mg (0.5–1.5 h) or 20 mg (2–3 h) embryos of indicated genotypes per replicate were hand selected, and dechorionated in 50% bleach for 5 minutes, fixed for 20 minutes in fixation buffer (50 mM HEPES, pH 8.0, 1 mM EDTA, 0.5 mM EGTA, 100 mM NaCl and 1.8% formaldehyde), and then quenched with 125 mM glycine (final concentration) for 5 minutes at room temperature. Next, the embryos were resuspended in 4 ml buffer A (24 mM EDTA, pH 8.0, 75 mM NaCl and protease inhibitor cocktail). The resuspended embryos were transferred to a dounce tissue grinder and homogenized with loose strokes for 50 times. The homogenized mixture was then centrifuged at 1500 g for 10 minutes at 4°C. To maximally remove the cytosolic components, repeat the dounce homogenization and centrifugation steps again. The resulting pellet was washed twice with buffer A and collected as nuclear fractions. Next, the nuclear pellet was resuspended in 4 ml buffer B (10 mM Tris-HCl, pH 8.0, 0.2 mM EDTA, 0.1% Triton X-100 and protease inhibitor cocktail). It was homogenized with tight strokes for 50 times and centrifuged at 1500 g for 10 minutes at 4°C. The pellet was washed twice with 1 ml of buffer B and centrifuged at 12000 g for 10 minutes at 4°C. The pellet was resuspended in 1 ml of buffer C (10 mM Tris-HCl, pH 8.0, 0.2 mM EDTA and protease inhibitor cocktail) and added to the top of 11 ml buffer D (10 mM Tris-HCl, pH 8.0, 1.7 M sucrose and 0.2 mM EDTA). The chromatins were collected by centrifugation at 70000 g for 3 hours at 4°C. Finally, the chromatins were washed twice with 1 ml buffer B by centrifugation at 20000 g for 20 minutes at 4°C, and stored at -80°C for further use.

Recombinant protein expression and purification

For the preparation of antibodies, *in vitro* binding assays, and phase separation assays, all recombinant proteins were expressed and purified from *Escherichia coli* BL21(DE3). In brief, an MBP, GST, or GFP tag was fused to the N-terminus of the target proteins in the constructs. BL21 cells were cultured in LB medium supplemented with 50 μg/ml kanamycin at 37°C until an optical density (OD_{600}) of 0.6 was reached. The cells were then induced with 0.5 mM IPTG and incubated for 18 hours at 16°C. After harvesting the cells by centrifugation at 1500 g for 5 minutes at 4°C, they were resuspended in a lysis buffer (20 mM Tris-Cl, pH 8.0, 5 mM imidazole, 500 mM NaCl and 1% Triton X-100). The cells were lysed using sonication and centrifuged at 16000 g for 20 minutes. The supernatants were collected and purified using Ni Sepharose™ High-Performance beads. The beads were washed twice with wash buffer 1 (20 mM Tris-Cl, pH 8.0, 20 mM imidazole and 500 mM NaCl) and once with wash buffer 2 (20 mM Tris-Cl, pH 8.0, 50 mM imidazole and 500 mM NaCl). The recombinant proteins were eluted with an elution buffer (20 mM Tris-Cl, pH 8.0, 500 mM imidazole, 500 mM NaCl and 10% glycerol). All protein purification steps were performed at 4°C. The concentration of recombinant proteins was determined using a BCA Protein Assay Kit (23227, Thermo Fisher).

In vitro binding assay between recombinant proteins

10 µg of recombinant His-GFP or His-GFP-dPCIF1 were incubated with Anti-GFP nanobody agarose in lysis buffer (50 mM Tris-HCl, pH 7.4, 1 mM EDTA, 150 mM NaCl, 10% glycerol and 1% NP-40) for 1 hour at 4°C. Following the incubation, the beads were washed three times with lysis buffer. The beads were then incubated with 10 µg of His-MBP-GAF for 2 hours, and again washed three times with lysis buffer at 4°C. The immunoprecipitated complexes were denatured with SDS-PAGE sample buffer, separated by SDS-PAGE, and subsequently analyzed by western blotting.

Identification of interacting proteins by mass spectrometry

Embryos of the specified genotypes were harvested, and total lysates were prepared in three biological replicates using a lysis buffer containing 50 mM Tris-HCl (pH 7.4), 1% NP-40, 150 mM NaCl, 1 mM EDTA, 10% glycerol and protease inhibitors. Samples of the total lysates (10 mg of protein per sample) were then subjected to immunoprecipitation using either a rabbit anti-GFP antibody (1:100), a mouse anti-GAF antibody (1:100), or normal IgG, followed by overnight incubation at 4°C. The mixtures were subsequently incubated with Pierce™ protein A/G agarose for 4 hours at 4°C. After incubation, the beads were washed three times with lysis buffer at 4°C, and the complexes were eluted by incubating the beads at 95°C for 10 minutes.

For LC-MS/MS analysis, the eluted complexes were separated using 10% SDS-PAGE until a migration distance of 0.5–1 cm was achieved from the stacking gel. The gels were then lightly stained with Coomassie Brilliant Blue, excised, and subjected to tryptic digestion. The resulting tryptic peptides were analyzed using the Orbitrap Exploris 480 MS system (Thermo Scientific). The MS/MS data were processed using Thermo Proteome Discoverer (version 2.4.0.305), and the tandem mass spectra were searched against the UniProt-*Drosophila* database UP000000803 (22075 entries) to identify unique protein sequences. Proteins consistently identified in all three replicates were included in subsequent analyses. We utilized abundance ratios to identify significantly altered proteins. Proteins with more than a 1.8-fold increase (q-value < 0.05) in three biological replicates compared to control IgG were considered dPCIF1/GAF-associated proteins.

ChIP-seq sample processing, library preparation and sequencing

50 mg embryos of indicated genotypes at the 0.5–1.5 hour stage per replicate (two biological replicates) were hand selected, dechorionated in 50% bleach for 5 minutes, fixed for 20 minutes in fixation buffer (50 mM HEPES, pH 8.0, 1 mM EDTA, 0.5 mM EGTA, 100 mM NaCl and 1.8% formaldehyde), and then quenched with 125 mM glycine (final concentration) for 5 minutes at room temperature. Subsequently, the fixed samples were resuspended in cell lysis buffer (5 mM HEPES, pH 8.0, 85 mM KCl, 0.5% IGEPAL CA-630 and protease inhibitors) and homogenized in a dounce homogenizer on ice by applying 50 strokes with the tight-fitting pestle, followed by centrifugation at 2000 g for 5 minutes to pellet the nuclei at 4℃. The subsequent steps were performed using the Pierce Magnetic ChIP Kit (26157, Thermo Fisher) according to

the manufacturer's instructions. The following antibodies were used: mouse anti-dPCIF1 (1:100), mouse anti-GAF (1:100), mouse anti-Zelda (1:100), mouse anti-HIRA (1:100), rabbit anti-RNA polymerase II-phospho S5 (1:200, ab5131, Abcam). Sequencing libraries were generated using the VAHTS Universal DNA Library Prep Kit for MGI (NDM607, Vazyme), and high-throughput sequencing was performed using the MGISEQ-2000RS platform.

Embryo staining and fluorescence analysis

For immunohistochemistry, embryos were fixed in a solution containing 4% formaldehyde and 0.3% Tween-20 in PBS for 30 minutes, followed by a 15-minute wash in PBT (0.3% Tween-20 in PBS). The fixed samples were then incubated overnight at 4°C with primary antibodies: mouse anti-dPCIF1 (1:1000), rabbit anti-RNA polymerase II-phospho S5 (1:2000, ab5131, Abcam), mouse anti-GAF (1:2000), rabbit anti-GAF (1:5000), mouse anti-HIRA (1:2000), or rabbit anti-H3K27ac (1:2000, C15410196, Diagenode). After incubation, the samples were washed three times and then incubated with the secondary antibody at room temperature for 2 hours, followed by three additional washes (10 minutes each) in PBT. Granule size was calculated using Imaris 9.0.1. Colocalization signals were assessed using the "Colocalization" module of Imaris 9.0.1, and the percentage of colocalization (% colocalization, or 'Intensity Weighted Overlap' statistic) was determined by calculating the percentage of summed pixel intensities of colocalization signals coinciding with GAF values above the threshold.

ATAC-seq sample processing, library preparation and sequencing

200 (0.5–1.5 h) or 30 (2–3 h) embryos of indicated genotypes per replicate (two biological replicates) were hand selected, and dechorionated in 50% bleach for 5 minutes. Subsequently, the embryos were resuspended with ATAC-lysis buffer (10 mM Tris-HCl, pH 7.4, 3 mM $MgCl_2$, 10 mM NaCl and 0.1% NP-40) and homogenized with a loose stroke 50 times, followed by centrifugation at 500 g for 5 minutes to pellet the nuclei at 4℃. The subsequent library preparation were performed using the TruePrep DNA Library Prep Kit for MGI (TDM501, Vazyme) according to the manufacturer's instructions, and high-throughput sequencing was performed using the MGISEQ-2000RS platform.

Octamer and nucleosome reconstitution

Histone octamers were prepared essentially as previously described (3). Briefly, the Fly H2A, H2B, H3, H3.3 and H4 proteins were expressed in *E. coli* and purified by cation and anion exchange. Equal molar amounts of individual histones were dissolved from lyophilized pellets in unfolding buffer (20 mM Tris-HCl, pH 7.5, 6 M guanidine hydrochloride and 5 mM DTT), and dialyzed overnight against several buffer changes of refolding buffer (10 mM Tris-HCL, PH 7.5, 1 mM EDTA, 2 M NaCl and 5 mM 2-mercaptoethanol). Precipitates were removed by centrifugation and octamers were further purified through a size exclusion chromatography

column. Elution fractions were analyzed by SDS-PAGE. The nucleosome assembly was performed using the microscale reconstitution method as previously described (4). The sequence of the DNA template is as follow:
GAATGAATGAACGAGAGGCGCCCACCCCGATAAACTTAAACTGAACGAACACTCAAGAG AGAGCGCAAGAGCGCTCAAAAACAATCTGGTTTTGAGCGTTTCGCTGGCTCTCTGTTTCT GTTTTCCACTCGTTTTTAGGCCGAGTCGAGTGAGTT. DNA were mixed with histone octamers at molar ratios of 1:1, and performed serial dilution using 10 mM Tris-HCl (pH 7.6).

SDD-AGE assay

The indicated proteins were expressed in *E. coli* and purified as described above. The proteins were then diluted with loading buffer (0.5xTBE, 10% glycerol, 2% SDS, 0.0025% bromophenol blue) at room temperature for 30 minutes, and loaded onto a newly-prepared 1.5% agarose gel with 0.1% SDS. After electrophoresis in the running buffer (1xTBE and 0.1% SDS) for 40 minutes with a constant voltage of 100 V at 4°C, the proteins were transferred to Immobilon membrane BioTrace NT nitrocellulose for immunoblotting with indicated antibodies.

Phase separation assay

The recombinant proteins were expressed and purified from *E. coli*. A glass bottom cell culture dish was utilized to generate the phase separation. For the nucleosome-dependent droplet-formation experiments, proteins were incubated with two types of nucleosomes containing NCP(H3) or NCP(H3.3) in the buffer (20 mM Tris-HCl, pH 7.4, 150 mM NaCl). Subsequently, the samples were immediately examined under Zeiss LSM 980 confocal microscopy, and images were captured at the indicated time points. The protein concentrations used in this experiment are specified in their figure legends. The percent of droplet area was calculated with the analyze particle tools in ImageJ from three replicates.

FISH probes prepare and DNA FISH

FISH probes for the *Ubx* promoter, enhancer, and the *Abd-B* promoter were prepared using the FISH Tag™ DNA Multicolor Kit (26157, Thermo Fisher). Specifically, $(AAGAG)_7$ DNA probes tagged with 5'Quasar 670 dye were custom-manufactured by HIPPO Biotechnology Company. DNA FISH was performed as described in a previous study (5). Briefly, the embryos at different stages were dechorionated with 50% sodium hypochlorite and fixed with 4% formaldehyde in the presence of heptane. The embryos were sequentially dehydrated and rehydrated in methanol/PBT buffer. The embryos were then treated with RNaseA at the concentration of 100-200 ug/ml in PBT for 2 hours at room temperature. The embryos were further treated with pre-Hybridization Mixture (PHM: 50% formamide, 4xSSC, 100 mM NaH_2PO_4, pH 7.0 and 0.1% Tween-20). After that, the embryo genomic DNA were denatured in PHM at 80°C for 15 minutes and the probes were denatured at 85°C for 10 minutes in FISH Hybridization buffer (FHB: 10% dextransulfat, 50% deionized formamide, 2xSSC and 0.5 mg/ml Salmon Sperm DNA). Then

remove the PHM and add the probe without colling. DNA hybridization was performed at 37°C for 20 hours. Following hybridization, embryos were washed with Formamide/SSC and Formamide/PBT buffers in sequence. For subsequent immunostaining, embryos were blocked in PBTA (PBS with 1.5% BSA and 0.3% Tween-20) for 1 hour at room temperature, then incubated with the primary antibody in PBTA buffer overnight at 4°C. After a series of washes, the embryos were incubated with the secondary antibody in PBTA buffer for 2 hours at room temperature. Finally, after three additional washes, the embryos were mounted and readied for confocal microscopy analysis.

PhosTag SDS-PAGE and lambda phosphatase treatment

Phosphorylation of dPCIF1 was analyzed by PhosTag SDS–polyacrylamide gel electrophoresis as described previously (6). The embryos of indicated stages were harvested and the total lysates were prepared with lysis buffer (50 mM Tris-HCl, pH 7.4, 1% NP-40, 150 mM NaCl, 10% glycerol and protease inhibitors). Proteins were separated on 8% SDS-PAGE gels supplemented with 50 mM PhosTag AAL (304-93521, Wako) and 50 mM $MnCl_2$. Mn^{2+} was chelated from gels by 10 minutes washes in transfer buffer supplemented with 1 mM EDTA. After three rinses in transfer buffer, standard immunoblotting was subsequently performed to detect dPCIF1. To dephosphorylate dPCIF1, the lysate of embryos was incubated with lambda phosphatase (P0753, New England Biolabs) in the presence of 1 mM $MnCl_2$ at 30°C for 30 minutes, and analyzed by PhosTag electrophoresis and immunoblotting.

Mapping the dPCIF1 phosphorylation sites by LC-MS/MS

Total embryonic proteins were used to map the dPCIF1 phosphorylation sites by LC-MS/MS. For tryptic digestion, desalting over C18 and enrichment of phosphopeptides over titanium dioxide beads was performed as described (7). 500 µg of total protein were predigested for 3 hours with endoproteinase Lys-C (0.5 µg/µl) at room temperature. After a 4-fold dilution with 10 mM Tris-HCl (pH 8.0), samples were digested overnight at 37°C with sequencing-grade modified trypsin (0.5 µg/µl). Digested peptides were mixed with an equal amount of isopropanol and enrichment buffer (1M glycolic acid in 80% v/v ACN and 6% v/v TFA) before phosphopeptide enrichment using titanium dioxide (TiO_2) beads (GL Sciences). The dissolved peptides were mixed with TiO_2 beads for 30 minutes, after which the beads-peptides mixture was washed twice with 80% v/v ACN and 1% v/v TFA. Phosphopeptides were eluted from the TiO_2 beads three times with a 1% v/v ammonia solution. The eluates were acidified and desalted over C18 stage tips (8). Peptide mixtures were analyzed using a nanoflow Easy-nLC (Thermo Scientific) and Orbitrap Exploris 480 MS system. Peptides were eluted from a 75 µm x 25 cm house-made analytical C18 column on a linear gradient running from 5% to 90% acetonitrile over 135 minutes. Proteins were identified based on the information-dependent acquisition of fragmentation spectra of multiply-charged peptides. Data-dependent MS/MS spectra were acquired in the linear ion trap for each full-scan spectrum acquired at a 70000 full-width at half-maximum (FWHM) resolution.

MaxQuant version 1.6.4.0 was used for raw file peak extraction and protein identification against the UniProt-Drosophila database (UP000000803, 22075 entries). The following parameters were applied: trypsin as the cleaving enzyme, with a minimum peptide length of seven amino acids and a maximum of two missed cleavages. Carbamidomethylation of cysteine was set as a fixed modification, while oxidation of methionine and phosphorylation of serine, threonine, and tyrosine were set as variable modifications. The peptide mass tolerance was set to 20 ppm, and the MS/MS tolerance was set to 0.5 Da. Additionally, peptide and protein false discovery rates (FDR) were set to 0.01, with common contaminants excluded.

RNA-seq data analysis

RNA-seq reads were trimmed by trimmomatic version 0.39 (9) and then mapped to *Drosophila melanogaster* (dm6) genome by star version 2.7.10a (10). RSEM version 1.3.1 (11) was used to calculate reads counts. Differential expression analyses were performed with R package DESeq2 version 1.40.2 (12). Genes with p.adjust < 0.05 and $\log_2$ foldchange >1 were considered differentially expressed. To identify potential target genes, ChIP peaks associated with GAF (13) and Zelda (14) were assigned to the nearest genes. Minor/major ZGA genes (15) and maternal genes (16) were previously defined.

ATAC-seq and ChIP-seq data processing

Paired-end reads were trimmed by trimmomatic version 0.39 (9) and then mapped to *Drosophila melanogaster* (dm6) using bowtie2 version 2.2.5 (17). PCR duplicates were marked using sambamba version 0.8.2 (18). Non-uniquely mapped reads, low-quality reads (quality scores less than 30) were removed using samtools version 1.18 (19). Bigwig files were generated from resulting deduplicated and filtered bam files using deeptools version 3.5.1 (20). Genomic tracks were visualized from 1x normalized bigWig files using Integrated Genome Viewer (21). Heatmaps of Reads Per Genomic Content (RPGC) were performed on indexed bam files using deeptools version 3.5.1 (20).

Peak analysis

Peaks were calculated using macs2 version 2.2.7.1 (22) with a q-value cutoff of 0.01. All downstream analyses focused on high-quality peaks that were detected in both replicates and overlapped by at least 20%. Peaks were assigned to either promoter or enhancer categories by identifying peaks that overlap known genomic annotations. Peaks that overlapped with the 1kb region flanking the transcriptional start site (TSS) were classified as promoters. The remaining peaks that overlapped with H3K27ac ChIP peaks (23) were classified as enhancer. The overlap of regions was performed using the R package GenomicRanges version 1.52.0 (24). Motif analyses were carried out with HOMER version 4.11.1 (25) and findMotifsGenome.pl was applied to enrich known motifs.

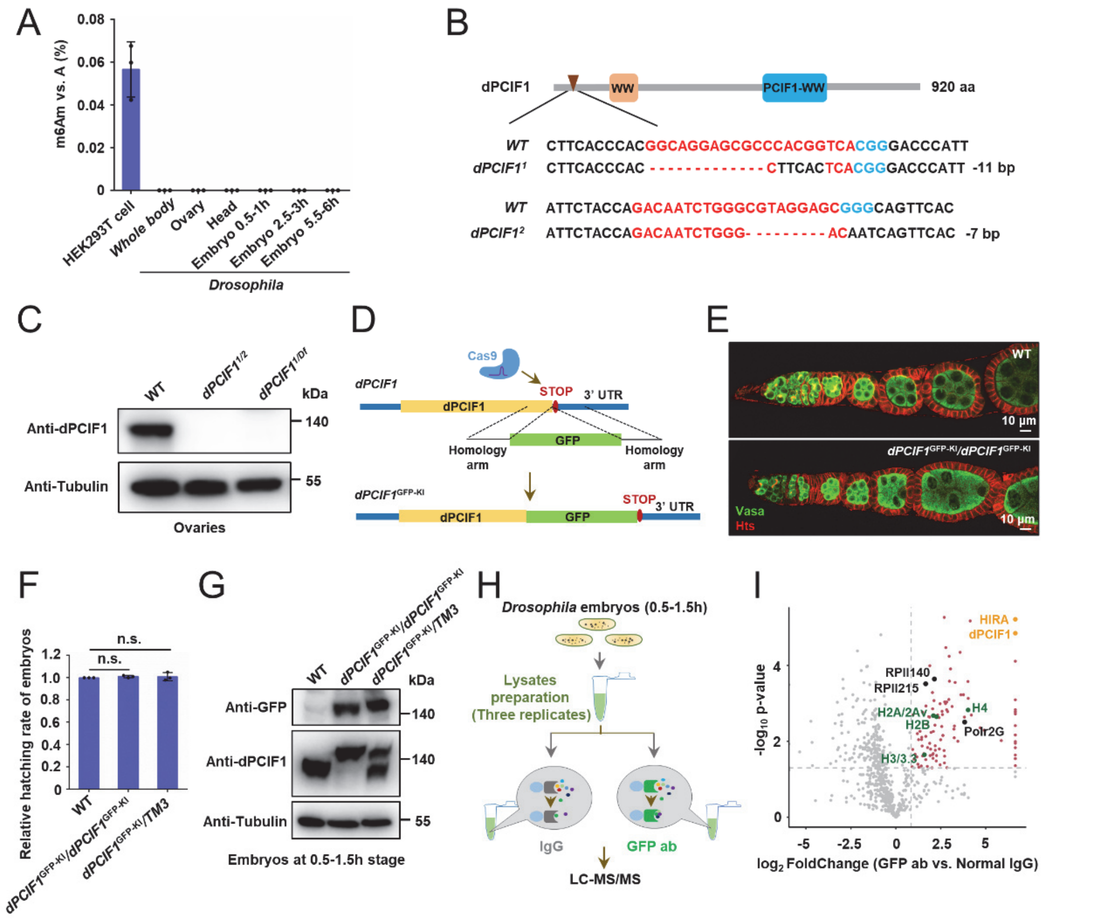

Fig. S1. dPCIF1 acts as a chromatin-associated factor regulating *Drosophila* embryogenesis.

(*A*) Bar plot showing the relative abundance of m6Am in HEK293T cells and in indicated samples from *Drosophila*. (*B*) Schematic representation of *dPCIF1* mutant allele generation using the CRISPR/Cas9 system. (*C*) Western blot shows that the dPCIF1 protein was completely abolished in *dPCIF1* mutant ovaries. (*D*) Schematic representation of dPCIF1-GFP knock-in allele (*dPCIF1*$^{\text{GFP-KI}}$) using the CRISPR/Cas9 system. (*E*) Immunostaining showing a normal oogenesis in *dPCIF1*$^{\text{GFP-KI}}$ homozygous ovaries. Scale bars, 10 μm. (*F*) Bar plot showing relative hatching rate of homozygous *dPCIF1*$^{\text{GFP-KI}}$ embryos. (*G*) Western blot shows that GFP knock-in does not change the level of endogenous dPCIF1 in *dPCIF1*$^{\text{GFP-KI}}$ flies. (*H*) Schematic representation of protocol for capturing dPCIF1-interacting proteins. (*I*) Volcano plot showing dPCIF1-interacting proteins in *dPCIF1*$^{\text{GFP-KI}}$ embryos at the 0.5–1.5 hour stage. Error bars indicate mean ± S.D (n = 3). n.s., not significant.

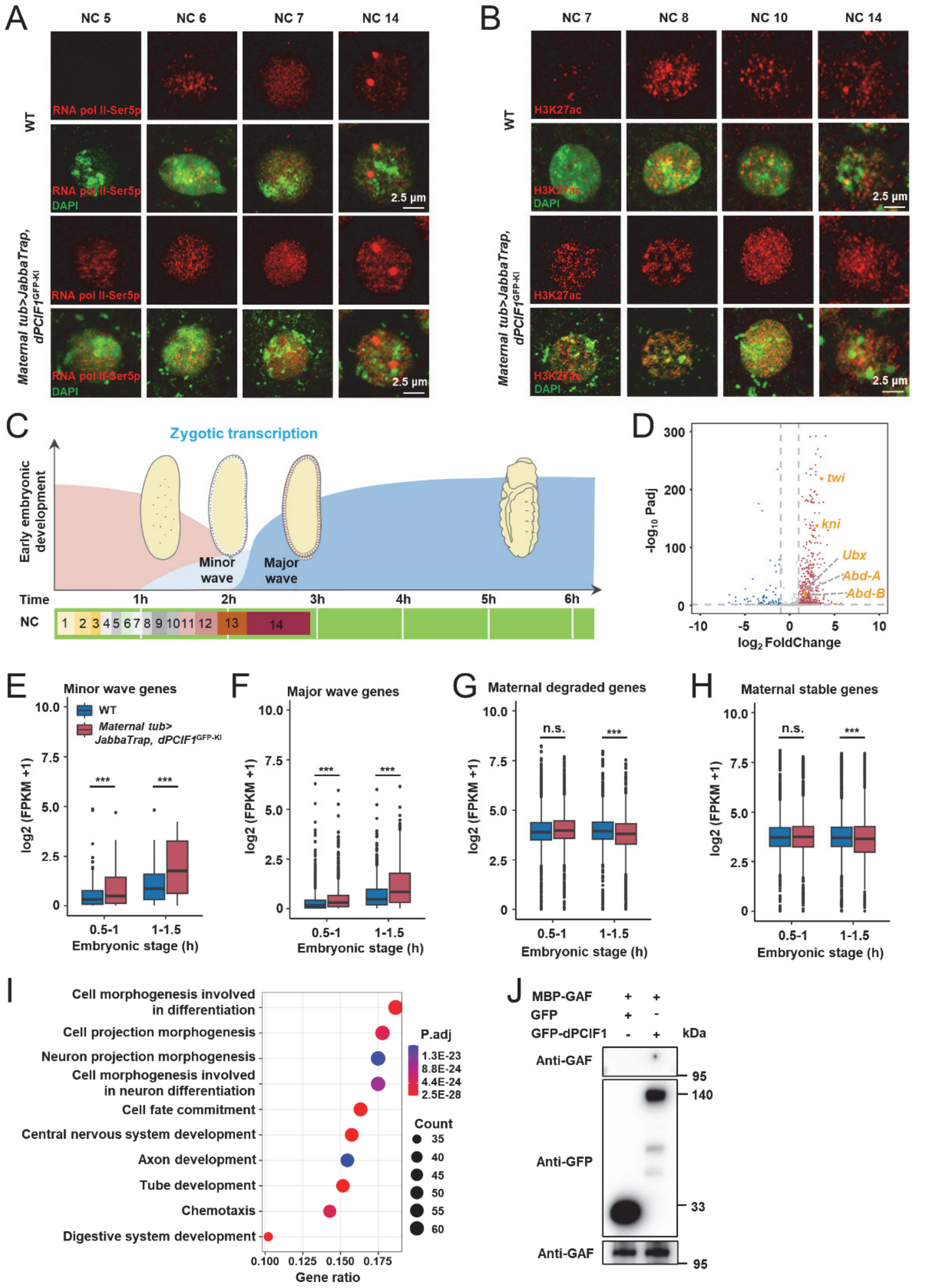

Fig. S2. dPCIF1 antagonizes GAF function to maintain normal ZGA.

(*A* and *B*) Immunostaining showing the signals of RNA Pol II-Ser5p (*A*) and H3K27ac (*B*) in wild-type and mis-localized dPCIF1 mutant embryos at indicated stages. Scale bars, 2.5 μm. (*C*) Schematic representation of minor- and major- wave zygotic gene activation over developmental time and nuclear division cycles. (*D*) Volcano plot showing differentially expressed genes between wild-type and mis-localized dPCIF1 mutant embryos at the 1–1.5 hour stage. (*E–H*) Box plot showing comparable expression levels of minor wave genes (*E*), major wave genes (*F*), maternal degraded genes (*G*) or maternal stable genes (*H*), between

wild-type and mis-localized dPCIF1 mutant embryos at indicated stages. *** $p < 0.001$, n.s., not significant. (*I*) Gene Ontology (GO) enrichment items of 458 major-wave zygotic genes that were ectopically expressed at the 1–1.5 hour stage. (*J*) *In vitro* pull-down assay showing no interaction between dPCIF1 and GAF.

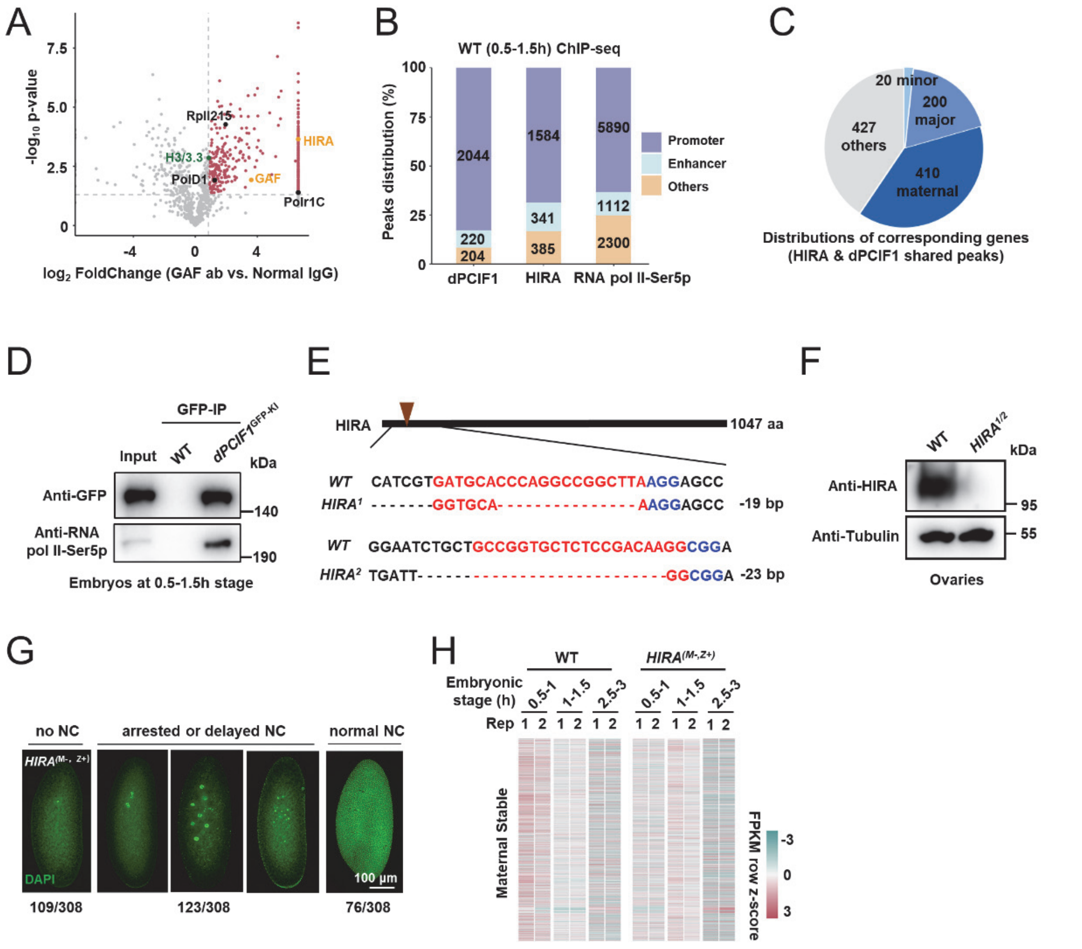

Fig. S3. dPCIF1 binds HIRA to restrict GAF function to maintain proper ZGA.

(*A*) Volcano plot showing GAF-interacting proteins in wild-type embryos at the 2–3 hour stage. (*B*) Bar plot showing the proportions of distinct genomic regions occupied by ChIP peaks in wild-type embryos. (*C*) The proportion of target genes that 60% shared peaks (HIRA & dPCIF1). (*D*) Co-IP showing the interaction of dPCIF1-GFP with RNA Pol II-Ser5p in *dPCIF1*$^{GFP-KI}$ embryos. (*E*) Schematic representation of *HIRA* mutant allele generation using the CRISPR/Cas9 system. (*F*) Western blot shows that the HIRA protein was completely abolished in *HIRA* mutant ovaries. (*G*) Maternal loss of HIRA showed arrested or delayed nuclear divisions. The proportions of embryos arrest in each stage are as indicated (n = 308). Scale bars, 100 µm. (*H*) Heatmap showing expression of maternal stable transcripts in wild-type and *HIRA* maternal mutant embryos at three different stages.

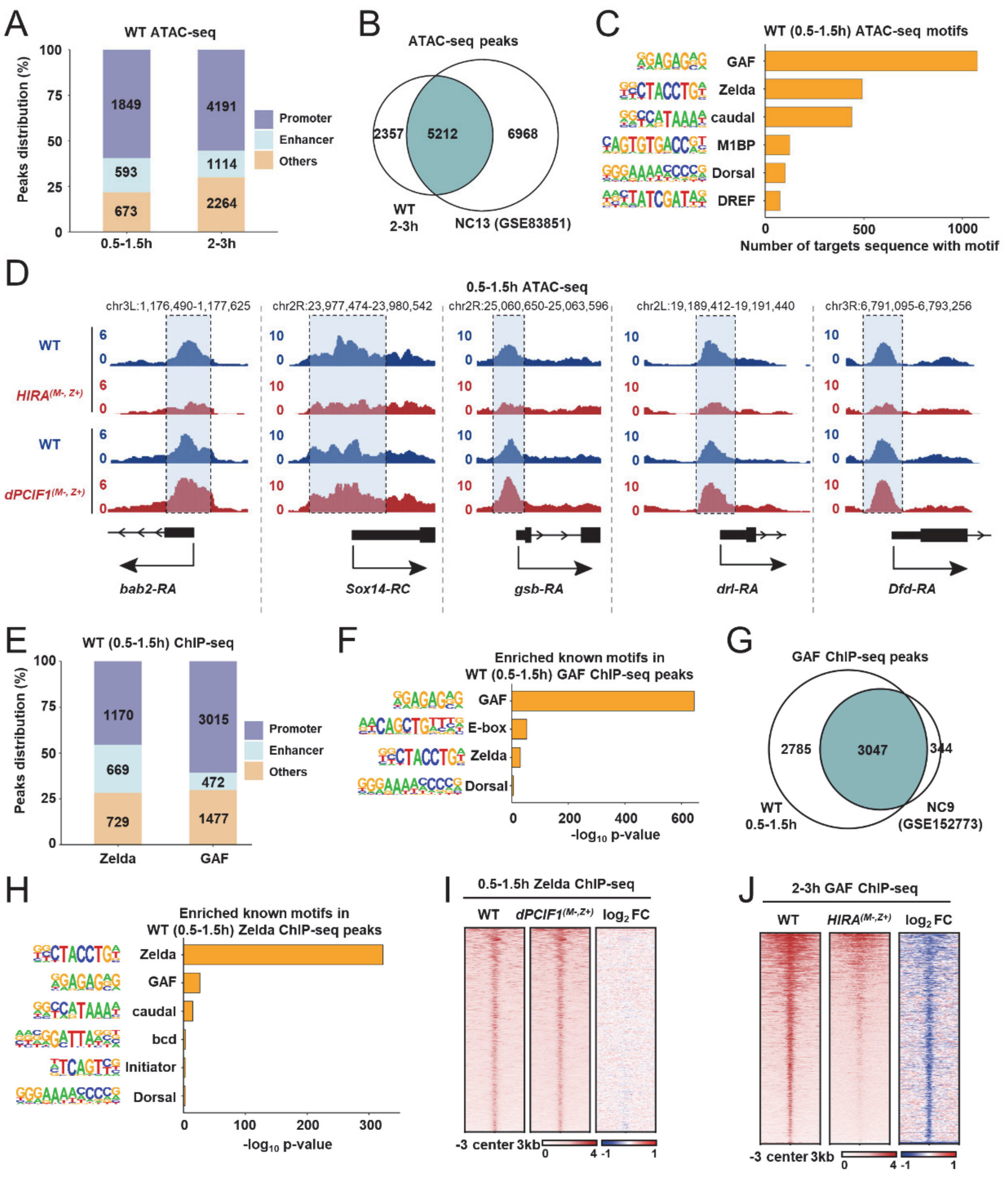

Fig. S4. HIRA is critical for pioneer factors to access chromatin.

(*A*) Bar plot showing the proportions of distinct genomic regions occupied by ATAC peaks in wild-type embryos. (*B*) Venn diagram showing the overlapping between our ATAC-seq dataset from wild-type embryos and published dataset from wild-type embryos (GSE83851). (*C*) Bar plot showing number of accessible chromatin peaks which contained GAF-, Zelda-, Caudal-, M1BP-, Dorsal-, and DREF-binding motifs in wild-type embryos. (*D*) IGV diagram displaying ATAC-seq signals on selected genes in the 0.5–1.5 hour embryos with indicated genotypes. (*E*) Bar plot showing the proportions of distinct genomic regions occupied by ChIP peaks in wild-type embryos. (*F*) Significant known motifs enriched in GAF ChIP-seq peaks from wild-type embryos. (*G*) Venn diagram showing the overlapping between our GAF ChIP-seq dataset from wild-type embryos and published dataset (GSE152773). (*H*) Significant known motifs enriched in Zelda ChIP-seq peaks from wild-type embryos. (*I*) Heatmap showing Zelda occupancy in wild-type and *dPCIF1* maternal mutant embryos. (*J*) Heatmap showing GAF occupancy in wild-type and *HIRA* maternal mutant embryos.

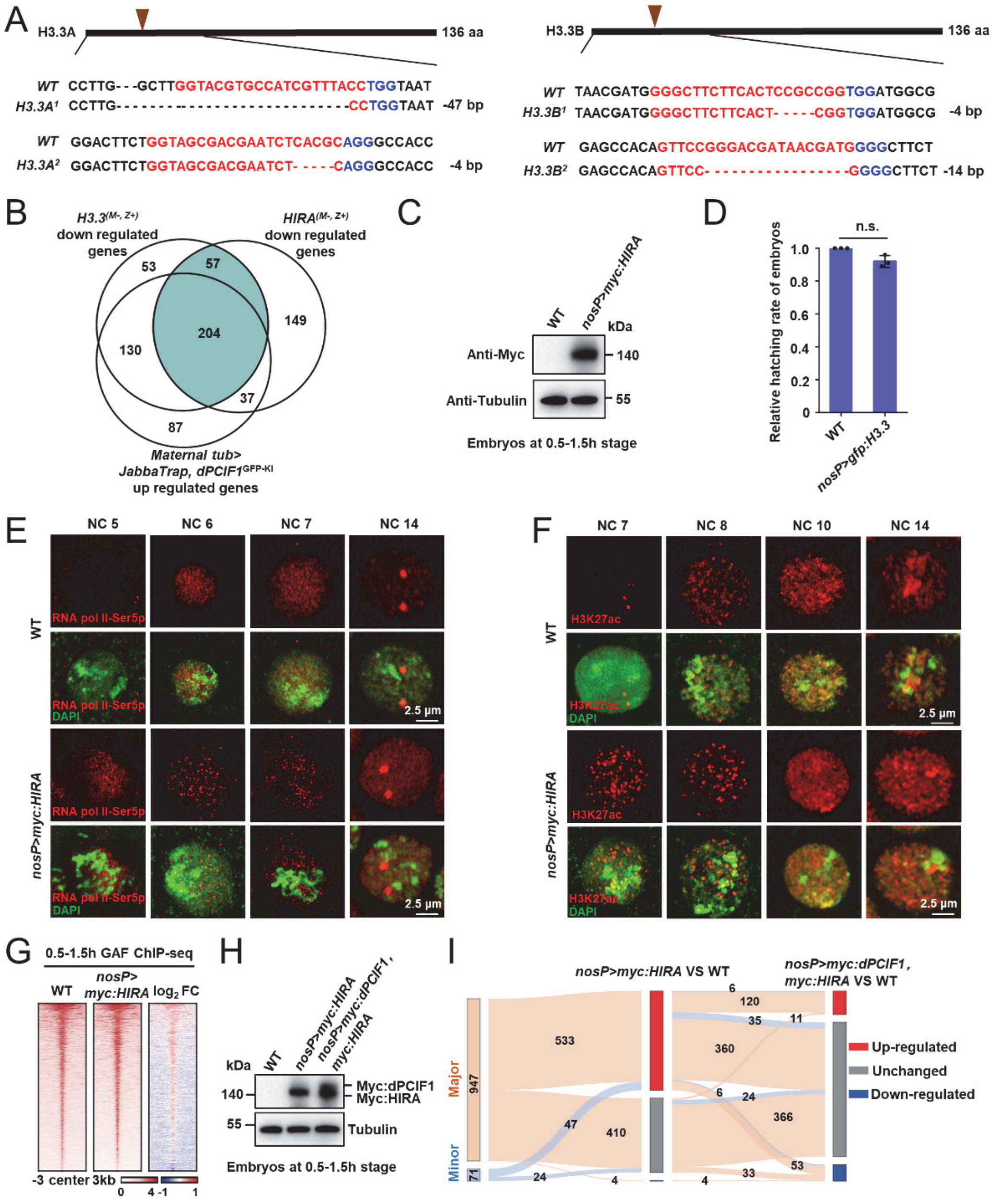

Fig. S5. HIRA and H3.3 coordinate to establish the totipotent state of early chromatin. (*A*) Schematic representation of *H3.3A* and *H3.3B* mutant allele generation using the CRISPR/Cas9 system. (*B*) Venn diagram showing overlapping of three gene categories. (*C*) Western blot showing the expression of Myc-HIRA protein in wild-type and maternal overexpressed HIRA embryos. (*D*) Bar plot showing the relative hatching rate of maternal overexpressed H3.3 embryos. Error bars indicate mean ± S.D (n=3). n.s., not significant. (*E* and *F*) Immunostaining showing the signals of RNA Pol II-Ser5p (*E*) and H3K27ac (*F*) in wild-type and maternal overexpressed HIRA embryos at indicated stages. Scale bar, 2.5 µm. (*G*) Heatmap showing GAF occupancy in wild-type and maternal overexpressed HIRA embryos. (*H*) Western blot showing the expression of Myc-HIRA and dPCIF1 proteins in the 0.5–1.5 hour embryos with indicated genotypes. (*I*) Sankey diagram showing ectopic gene expression in the 1–1.5 hour embryos with indicated genotypes.

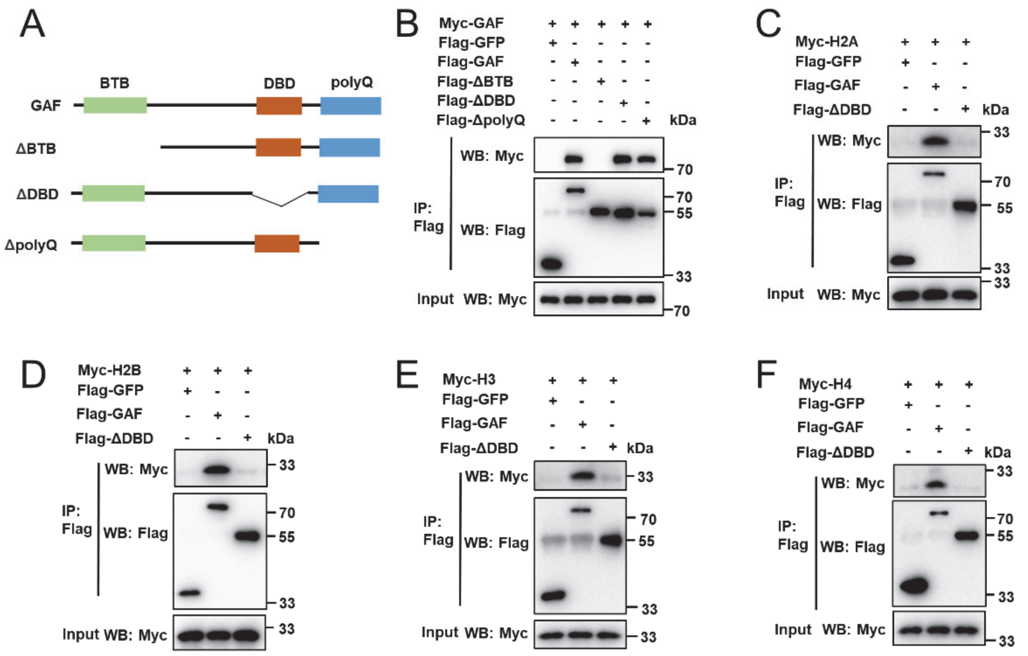

Fig. S6. GAF was associated with histone proteins through its DBD doman.
(*A*) Schematic representation of GAF domain architecture and truncations. (*B*) Co-IP showing the interaction of Myc-GAF with Flag-tagged full-length or truncated GAF in S2 cells. (*C–F*) Co-IP showing the interaction of Flag-GAF with Myc-tagged histone proteins in S2 cells.

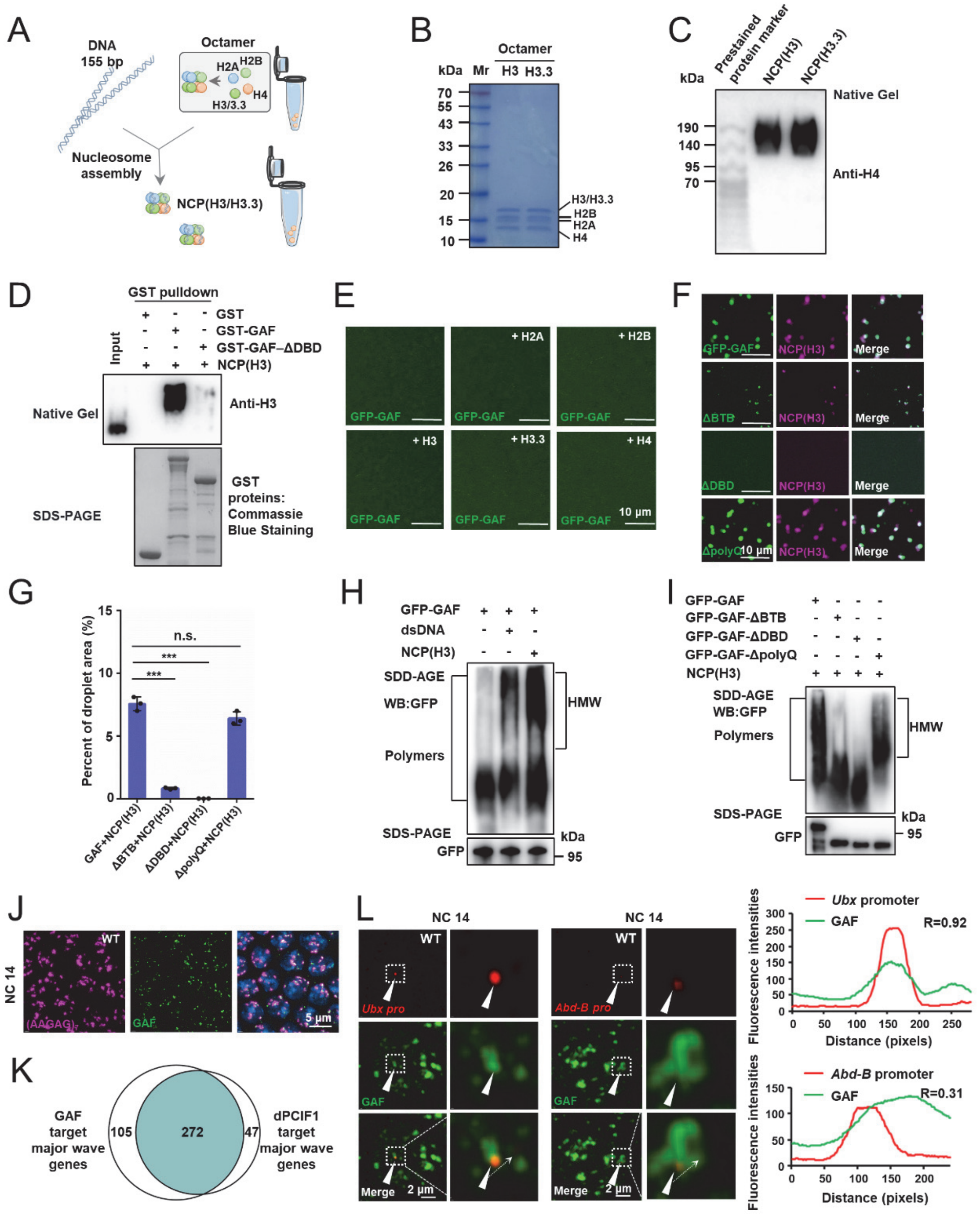

Fig. S7. Nucleosome binding promotes condensation of GAF that contributes to ZGA. (*A*) Schematic representation of *in vitro* nucleosome assembling process. (*B*) SDS-PAGE analysis showing octamer assembled by histones purified from bacteria. (*C*) Native PAGE showing assembled NCP(H3) and NCP(H3.3) marked by H4 antibody. (*D*) *In vitro* pull-down assays showing lack of DBD domain abolishes the interaction between GAF and nucleosomes. (*E*) Droplet formation assays for GFP-GAF (1 mg/ml) mixed with various histone proteins (0.2 mg/ml). Scale bars, 10 µm. (*F*) Droplet formation assays for GFP-GAF (1 mg/ml), ΔBTB (1 mg/ml), ΔDBD (1 mg/ml) and ΔpolyQ (1 mg/ml) mixed with NCP(H3) (0.1 µM). Scale bars, 10 µm. (*G*) The quantitative area of green droplet signal in (*F*). *** $p < 0.001$, n.s., not significant. (*H*) Semi-denaturing Detergent Agarose Gel Electrophoresis (SDD-AGE) assay showing

nucleosomes promote GAF to condensate into high molecular weight oligomers. (*I*) SDD-AGE assay showing GAF lacking BTB or DBD domain failed to form high molecular weight oligomers. (*J*) Immunostaining combining with DNA-FISH reveal that the majority of the GAF larger granules colocalize with the signal of the $(AAGAG)_7$ repeats. Scale bars, 5 μm. (*K*) dPCIF1 and GAF ChIP-seq datasets from wild-type and *dPCIF1* maternal mutant embryos showing 272 genes are both GAF and dPCIF1 targeted genes. (*L*) DNA-FISH showing GAF proteins co-localize with the FISH signals of *Ubx* and *Abd-B* gene promoter regions. Right panel shows the pearson's correlation coefficients of GAF and the promoter regions. Scale bars, 2 μm.

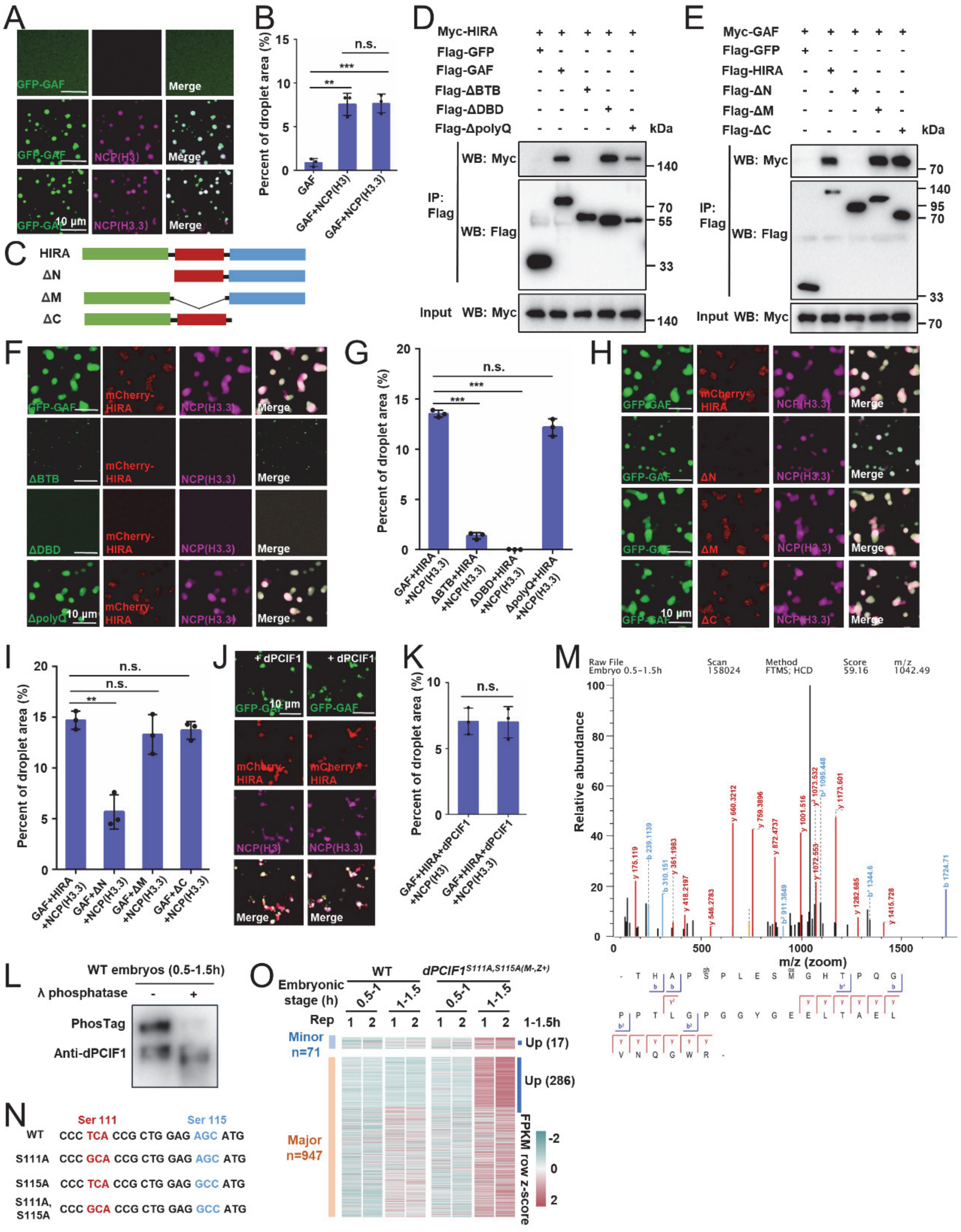

Fig. S8. HIRA and dPCIF1 coordinately determine the phase separation and function of GAF in early embryos.

(*A*) Droplet formation assays for GFP-GAF (1 mg/ml) mixed with NCP(H3 or H3.3) (0.1 μM). Scale bars, 10 μm. (*B*) The quantitative area of green droplet signal in (*A*). (*C*) Schematic representation of HIRA domain architecture and truncations. (*D*) Co-IP showing the interaction of Myc-HIRA with Flag-tagged full-length or truncated GAF in S2 cells. (*E*) Co-IP showing the interaction of Myc-GAF with Flag-tagged full-length or truncated HIRA in S2 cells. (*F*) Droplet formation assays for GFP-GAF (1 mg/ml), ΔBTB (1 mg/ml), ΔDBD (1 mg/ml) and ΔpolyQ (1

mg/ml) mixed with mCherry-HIRA (1 mg/ml) and NCP(H3.3) (0.1 μM). Scale bars, 10 μm. (*G*) The quantitative area of green droplet signal in (*F*). (*H*) Droplet formation assays for mCherry-HIRA (1 mg/ml), ΔN (1 mg/ml), ΔM (1 mg/ml) and ΔC (1 mg/ml) mixed with GFP-GAF (1 mg/ml) and NCP(H3.3) (0.1 μM). Scale bars, 10 μm. (*I*) The quantitative area of green droplet signal in (*H*). (*J*) Droplet formation assays for GFP-GAF (1 mg/ml) mixed with mCherry-HIRA (1 mg/ml), dPCIF1 (0.5 mg/ml) and NCP(H3 or H3.3) (0.1 μM). Scale bars, 10 μm. (*K*) The quantitative area of green droplet signal in (*J*). (*L*) Phos-tag gel electrophoresis assay showed that treatment of lambda protein phosphatase abolished the dPCIF1 band shift. (*M*) Mapping the dPCIF1 phosphorylation sites in the 0.5-1.5 hour embryos by LC-MS/MS. (*N*) Nucleotide sequences showing the mutation forms of dPCIF1^{S111A}, dPCIF1^{S115A}, and dPCIF1^{S111A,S115A}. (*O*) Heatmap showing expression of minor and major wave zygotic transcripts in wild-type and *dPCIF1*^{S111A,S115A} embryos at two different stages before major-wave ZGA. ** $p < 0.01$, *** $p < 0.001$, n.s., not significant.

Table S1: Primers for fly preparation and screening

Primer name	Primer sequence 5' - 3'
dPCIF1 target 1	TAATACGACTCACTATAGGGCAGGAGCGCCCACGGTCAG TTTTAGAGCTAGAAATAGC
dPCIF1 target 2	TAATACGACTCACTATAGGACAATCTGGGCGTAGGAGCGT TTTAGAGCTAGAAATAGC
dPCIF1-screen-F	ATGGCAGCTAACAACAAC
dPCIF1-screen-R	CATCTCCCACAGCGATTC
HIRA target 1	TAATACGACTCACTATAGGATGCACCCAGGCCGGCTTAGT TTTAGAGCTAGAAATAGC
HIRA target 2	TAATACGACTCACTATAGGCCGGTGCTCTCCGACAAGGGT TTTAGAGCTAGAAATAGC
HIRA-screen-F	TAGCGCCTATCGATGACGAT
HIRA-screen-R	GCATTTCCACGATTCGTGAT
H3.3A target 1	TAATACGACTCACTATAGGTACGTGCCATCGTTTACCGTTT TAGAGCTAGAAATAGC
H3.3A target 2	TAATACGACTCACTATAGGTAGCGACGAATCTCACGCGTT TTAGAGCTAGAAATAGC
H3.3A-screen-F	TTGACCTTATACGGAATACCC
H3.3A-screen-R	ACAGATTGGTGTCCTCGAACA
H3.3B target 1	TAATACGACTCACTATAGGGCTTCTTCACTCCGCCGGGTT TTAGAGCTAGAAATAGC
H3.3B target 2	TAATACGACTCACTATAGGTTCCGGGACGATAACGATGGT TTTAGAGCTAGAAATAGC
H3.3B-screen-F	TCTCATCTGGCGAGGATCAGC
H3.3B-screen-R	CAGGTACGCCTCAGATGCTTC
gRNA-R	AGCACCGACTCGGTGCCACT
Homology arm #1 of *dPCIF1*$^{GFP\text{-}KI}$-F	TCGCTGAAGCAGGTGGAATTTAAACCCTCCTCATTGCGAG
Homology arm #1 of *dPCIF1*$^{GFP\text{-}KI}$-R	GCCCTTGCTCACCATGACGGCTGAGTTGATGAC
Homology arm #2 of *dPCIF1*$^{GFP\text{-}KI}$-F	GAGCTGTACAAGTAACTGTTAGCATAAATTAGGGTTAC
Homology arm #2 of *dPCIF1*$^{GFP\text{-}KI}$-R	GCCGCTAGCATGCAAGAATTTGCCTACAAGCTCCTTGAAG
dPCIF1$^{GFP\text{-}KI}$ CRISPR/Cas target 1	TATATAGGAAAGATATCCGGGTGAACTTCGGACAAAATAG AACCATGTGGTTTTAGAGCTAGAAATAGCAAG
dPCIF1$^{GFP\text{-}KI}$ CRISPR/Cas target 2	ATTTTAACTTGCTATTTCTAGCTCTAAAACTACTATCTGTGC ACTTTCTGCGACGTTAAATTGAAAATAGGTC
Homology arm #1 of *dPCIF1*^{S111A,S115A}-F	TCGCTGAAGCAGGTGGTACTACTCCCCACGATCTCCA

Homology arm #1 of *dPCIF1*^{S111A,S115A}-R	ATGTGTTTTCGTGGGCGTCGAGGGGGAGGAGCAACT
Homology arm #2 of *dPCIF1*^{S111A,S115A}-F	TACGTTAGGTCCCGGTGGATATGGCGAGGAGCTAA
Homology arm #2 of *dPCIF1* ^{S111A,S115A}-F	CCGCTAGCATGCAAGTCCTGTAAGTACTCGATCGT
dPCIF1^{S111A}*-sequence*	GCCCACGAAAACACATGCGCCCGCACCGCTCGAGAGTAT GGGGCACACACCGCAAGGACCCCCTACGTTAGGTCCCGG
dPCIF1^{S115A}*-sequence*	GCCCACGAAAACACATGCGCCCTCACCGCTCGAGGCCAT GGGGCACACACCGCAAGGACCCCCTACGTTAGGTCCCGG
dPCIF1^{S115A,S115A}*-sequence*	GCCCACGAAAACACATGCGCCCGCACCGCTCGAGGCCAT GGGGCACACACCGCAAGGACCCCCTACGTTAGGTCCCGG
dPCIF1^{S111A,S115A} CRISPR/Cas target 1	TATATAGGAAAGATATCCGGGTGAACTTCGTATGGCCCAT GCTCTCCAGGTTTTAGAGCTAGAAATAGCAAG
dPCIF1^{S111A,S115A} CRISPR/Cas target 2	ATTTTAACTTGCTATTTCTAGCTCTAAAACGGTCCCAAGGT TGGCGGACCGACGTTAAATTGAAAATAGGTC

Table S2: Primers for FISH probe synthesis

Primer name	Primer sequence 5’ - 3’
Ubx-promoter-probe-s	CCTGGCTTCTATTACCGTA
Ubx-promoter-probe-as	GGTCGGATCTTCAGGACACT
Ubx-enhancer-probe-s	TGTGGTTCGACGTTTTCCCG
Ubx-enhancer-probe-as	GCTCAAAAGACGAGCTTACG
Abd-B-promoter-probe-s	GAGGATAAAGGCGGTGCAGT
Abd-B-promoter-probe-as	TGGAAATAGATTGCGGCAGT
$(AAGAG)_7$ probe	AAGAGAAGAGAAGAGAAGAGAAGAGAAGAGAAGAG

Table S3: Plasmids used in this study

Recombinant DNA	Source
Plasmid: uasp-Myc-dPCIF1	This paper
Plasmid: uasp-Myc-HIRA	This paper
Plasmid: uasp-GFP-H3.3	This paper
Plasmid: uasp-VhhGFP4-jabba-VhhGFP4	This paper
Plasmid: pet28a-H2A	This paper
Plasmid: pet28a-H2B	This paper
Plasmid: pet28a-H3	This paper
Plasmid: pet28a-H3.3	This paper
Plasmid: pet28a-H4	This paper
Plasmid: pet28a-His-H2A	This paper
Plasmid: pet28a-His-H2B	This paper
Plasmid: pet28a-His-H3	This paper
Plasmid: pet28a-His-H3.3	This paper
Plasmid: pet28a-His-H4	This paper
Plasmid: pet42a-GAF	This paper
Plasmid: pet42a-GAF$^{\Delta DBD}$	This paper
Plasmid: pet28a-GFP	This paper
Plasmid: pet28a-GFP-GAF	This paper
Plasmid: pet28a-GFP-GAF$^{\Delta BTB}$	This paper
Plasmid: pet28a-GFP-GAF$^{\Delta DBD}$	This paper
Plasmid: pet28a-GFP-GAF$^{\Delta polyQ}$	This paper
Plasmid: pet28a-mCherry-HIRA	This paper
Plasmid: pet28a-mCherry-HIRA$^{\Delta N}$	This paper
Plasmid: pet28a-mCherry-HIRA$^{\Delta M}$	This paper
Plasmid: pet28a-mCherry-HIRA$^{\Delta C}$	This paper
Plasmid: pet42a-dPCIF1	This paper
Plasmid: pet28a-GFP-dPCIF1	This paper
Plasmid: pet28a-MBP-dPCIF1$^{1\text{-}200}$	This paper
Plasmid: pet28a-MBP-GAF$^{52\text{-}202}$	This paper
Plasmid: pet28a-MBP-HIRA$^{501\text{-}700}$	This paper
Plasmid: pet28a-MBP-Zelda$^{351\text{-}721}$	This paper
Plasmid: pAC5.1-Flag-GAF	This paper
Plasmid: pAC5.1-Flag-GAF$^{\Delta BTB}$	This paper
Plasmid: pAC5.1-Flag-GAF$^{\Delta DBD}$	This paper
Plasmid: pAC5.1-Flag-GAF$^{\Delta polyQ}$	This paper
Plasmid: pAC5.1-Flag-HIRA	This paper
Plasmid: pAC5.1-Flag-HIRA$^{\Delta N}$	This paper
Plasmid: pAC5.1-Flag-HIRA$^{\Delta M}$	This paper
Plasmid: pAC5.1-Flag-HIRA$^{\Delta C}$	This paper
Plasmid: pAC5.1-Flag-dPCIF1	This paper

Plasmid: pAC5.1-Flag-dPCIF1^{S111A}	This paper
Plasmid: pAC5.1-Flag-dPCIF1^{S115A}	This paper
Plasmid: pAC5.1-Flag-dPCIF1 $^{S111A, S115A}$	This paper
Plasmid: pAC5.1-Myc-GAF	This paper
Plasmid: pAC5.1-Myc-HIRA	This paper
Plasmid: pAC5.1-Myc-H2A	This paper
Plasmid: pAC5.1-Myc-H2B	This paper
Plasmid: pAC5.1-Myc-H3	This paper
Plasmid: pAC5.1-Myc-H4	This paper

SI References

1. Zhang G*, et al.* (2015) N6-methyladenine DNA modification in *Drosophila*. *Cell* 161(4):893-906.
2. He S*, et al.* (2019) 6mA-DNA-binding factor Jumu controls maternal-to-zygotic transition upstream of Zelda. *Nature communications* 10(1):2219.
3. Klinker H, Haas C, Harrer N, Becker PB, & Mueller-Planitz F (2014) Rapid purification of recombinant histones. *PloS one* 9(8):e104029.
4. Dyer PN*, et al.* (2004) Reconstitution of nucleosome core particles from recombinant histones and DNA. *Methods in enzymology* 375:23-44.
5. Bantignies F & Cavalli G (2014) Topological organization of *Drosophila* Hox genes using DNA fluorescent in situ hybridization. *Methods in molecular biology* 1196:103-120.
6. Li T*, et al.* (2021) Phosphorylation and chromatin tethering prevent cGAS activation during mitosis. *Science* 371(6535).
7. Wu XN*, et al.* (2017) Highly Efficient Single-Step Enrichment of Low Abundance Phosphopeptides from Plant Membrane Preparations. *Frontiers in plant science* 8:1673.
8. Rappsilber J, Ishihama Y, & Mann M (2003) Stop and go extraction tips for matrix-assisted laser desorption/ionization, nanoelectrospray, and LC/MS sample pretreatment in proteomics. *Analytical chemistry* 75(3):663-670.
9. Bolger AM, Lohse M, & Usadel B (2014) Trimmomatic: a flexible trimmer for Illumina sequence data. *Bioinformatics* 30(15):2114-2120.
10. Dobin A*, et al.* (2013) STAR: ultrafast universal RNA-seq aligner. *Bioinformatics* 29(1):15-21.
11. Li B & Dewey CN (2011) RSEM: accurate transcript quantification from RNA-Seq data with or without a reference genome. *BMC Bioinformatics* 12:323.
12. Love MI, Huber W, & Anders S (2014) Moderated estimation of fold change and dispersion for RNA-seq data with DESeq2. *Genome biology* 15(12):550.
13. Gaskill MM, Gibson TJ, Larson ED, & Harrison MM (2021) GAF is essential for zygotic genome activation and chromatin accessibility in the early *Drosophila* embryo. *eLife* 10.
14. Harrison MM, Li XY, Kaplan T, Botchan MR, & Eisen MB (2011) Zelda binding in the early *Drosophila melanogaster* embryo marks regions subsequently activated at the maternal-to-zygotic transition. *PLoS genetics* 7(10):e1002266.

15. Riemondy K, Henriksen JC, & Rissland OS (2023) Intron dynamics reveal principles of gene regulation during the maternal-to-zygotic transition. *Rna* 29(5):596-608.
16. Zhang G*, et al.* (2022) Dynamic FMR1 granule phase switch instructed by m6A modification contributes to maternal RNA decay. *Nature communications* 13(1):859.
17. Langmead B & Salzberg SL (2012) Fast gapped-read alignment with Bowtie 2. *Nature methods* 9(4):357-359.
18. Tarasov A, Vilella AJ, Cuppen E, Nijman IJ, & Prins P (2015) Sambamba: fast processing of NGS alignment formats. *Bioinformatics* 31(12):2032-2034.
19. Danecek P*, et al.* (2021) Twelve years of SAMtools and BCFtools. *Gigascience* 10(2).
20. Ramírez F*, et al.* (2016) deepTools2: a next generation web server for deep-sequencing data analysis. *Nucleic acids research* 44(W1):W160-165.
21. Robinson JT*, et al.* (2011) Integrative genomics viewer. *Nature Biotechnology* 29(1):24-26.
22. Zhang Y*, et al.* (2008) Model-based analysis of ChIP-Seq (MACS). *Genome biology* 9(9):R137.
23. mod EC*, et al.* (2010) Identification of functional elements and regulatory circuits by *Drosophila* modENCODE. *Science* 330(6012):1787-1797.
24. Lawrence M*, et al.* (2013) Software for computing and annotating genomic ranges. *PLoS computational biology* 9(8):e1003118.
25. Heinz S*, et al.* (2010) Simple combinations of lineage-determining transcription factors prime cis-regulatory elements required for macrophage and B cell identities. *Molecular cell* 38(4):576-589.
